# LC–MS-Based Metabolomic Profiling of *Excoecaria agallocha* Leaf Extract: Integrated Antioxidant, Anti-Inflammatory, Cholinesterase Inhibitory and Neuroprotective Evaluation with AChE-Targeted Molecular Docking

**DOI:** 10.3390/molecules31203632

**Published:** 2026-10-12

**Authors:** Ayed A. Alshamrani

**Affiliations:** Department of Pharmacology and Toxicology, College of Pharmacy, Jazan University, Jazan 45142, Saudi Arabia; aashamrani@jazanu.edu.sa

**Keywords:** *Excoecaria agallocha*, antioxidant activity, COX-2 inhibition, acetylcholinesterase, neuroprotection, trimethyltin

## Abstract

*Excoecaria agallocha* L. is a medicinally important mangrove species containing diverse secondary metabolites with potential antioxidant, anti-inflammatory, and neuroprotective activities. This study investigated the multifunctional biological potential of *E. agallocha* leaves using petroleum ether, chloroform, ethyl acetate, methanol, and aqueous extracts. Preliminary phytochemical screening revealed a solvent-dependent distribution of constituents, while total phenolic and flavonoid contents were quantified in the methanolic and aqueous extracts. Antioxidant activity was evaluated using DPPH radical-scavenging and ferric-reducing antioxidant power (FRAP) assays, while anti-inflammatory activity was assessed using protein-denaturation and COX-2 inhibition assays, with aspirin and celecoxib as reference standards, respectively. Cytotoxicity of the methanolic and aqueous extracts was evaluated in L929 normal fibroblasts, and neuroprotective activity was assessed in SK-N-SH neuroblastoma cells exposed to trimethyltin (TMT), with the aqueous extract administered 1 h before TMT exposure. Apoptotic changes were evaluated using acridine orange/ethidium bromide (AO/EB) dual-fluorescence staining. Acetylcholinesterase (AChE) inhibition was determined using galantamine as the reference inhibitor. LC–MS was employed for putative metabolite profiling of the aqueous extract, followed by AChE-targeted molecular docking of selected identified compounds against human acetylcholinesterase (AChE; PDB ID 7E3D) and ProTox-II-based in silico toxicity prediction. The aqueous extract showed the highest total phenolic (717.81 µg/g) and flavonoid (485.16 µg/g) contents compared with the methanolic extract (562.43 and 334.00 µg/g, respectively) and demonstrated the strongest DPPH radical-scavenging activity among the extracts (IC_50_ = 115.49 µg/mL) together with a marked ferric-reducing capacity. The aqueous extract also exhibited pronounced COX-2 inhibition (IC_50_ = 39.77 µg/mL), whereas the methanolic extract showed greater inhibition of protein denaturation (IC_50_ = 138.89 µg/mL). The aqueous extract displayed relatively low cytotoxicity toward L929 cells and markedly attenuated TMT-induced toxicity in SK-N-SH cells, with the greatest protection observed at the lowest pre-treatment concentration (20 µg/mL; 92.14 ± 1.77% cell viability versus 16.92 ± 1.55% with TMT alone). AChE inhibition by the aqueous extract increased concentration-dependently, with an IC_50_ of 55.64 µg/mL. Consistent with these findings, AO/EB staining demonstrated an increased proportion of viable cells and reduced apoptotic cell populations following aqueous-extract pre-treatment. LC–MS profiling revealed a chemically diverse array of putatively identified metabolites. AChE-targeted molecular docking putatively identified 6-hydroxy-2-(4-methoxyphenyl)-4H-chromen-4-one and daidzein showed comparatively favorable docking profiles together with Class 5 predicted toxicity. Collectively, the present findings demonstrate that *E. agallocha* leaf extracts possess measurable antioxidant, anti-inflammatory, cholinesterase-inhibitory, and neuroprotective-related activities under the experimental conditions investigated, with the aqueous extract showing comparatively pronounced activity. These findings provide preliminary experimental and computational evidence supporting further bioassay-guided isolation, experimental validation of individual compounds, mechanistic studies, and in vivo evaluation to determine the neuroprotective potential and biological relevance of *E. agallocha*.

## 1. Introduction

Plants have historically served as an important source of therapeutic agents because their specialized metabolism produces structurally diverse molecules capable of interacting with multiple biological targets. Unlike single-molecule synthetic drugs, plant extracts contain complex mixtures of phenolics, flavonoids, terpenoids, alkaloids, sterols, lignans and related metabolites that may act through complementary antioxidant, anti-inflammatory, enzyme-modulating and cytoprotective mechanisms. This chemical diversity is particularly relevant to multifactorial disorders in which oxidative stress, inflammation, mitochondrial dysfunction and neuronal damage occur simultaneously. Polyphenols, for example, can influence both oxidative and inflammatory pathways and are increasingly investigated as multitarget candidates for neurodegenerative disease research [1].

Mangrove plants represent a particularly valuable but underexplored reservoir of natural products. Their growth under high salinity, tidal inundation, oxygen limitation, intense irradiation and other environmental stresses imposes strong selective pressure for the production of protective secondary metabolites. Consequently, mangroves contain diverse chemical classes, including flavonoids, phenolics, tannins, terpenoids, steroids, alkaloids and other specialized compounds with reported antioxidant, antimicrobial, anti-inflammatory, antidiabetic and cytoprotective activities. Reviews of mangrove pharmacology have emphasized their potential for natural-product discovery while also identifying the need for stronger experimental validation and systematic safety assessment [2].

Among medicinal mangroves, *Excoecaria agallocha* L. (Euphorbiaceae), commonly called the blind-your-eye mangrove, is particularly noteworthy. The species is distributed throughout tropical and subtropical coastal regions and has a long history of ethnomedicinal use for conditions including rheumatism, epilepsy, ulcers, paralysis and inflammatory disorders [3]. Its leaves, bark, stems and roots contain diverse phytochemicals, particularly diterpenoids, triterpenoids, flavonoids, sterols and phenolic constituents. Recent comprehensive evidence indicates that more than 235 phytoconstituents have been reported from *E. agallocha*, accompanied by antioxidant, anti-inflammatory, antimicrobial, anticancer, anticonvulsant and other pharmacological activities [4]. More specifically, diterpenoids isolated from *E. agallocha* have recently demonstrated anti-neuroinflammatory activity through modulation of macrophage polarization and inflammasome-associated pathways, further strengthening the therapeutic interest of this species [5].

Neurodegenerative diseases, including Alzheimer’s disease, Parkinson’s disease, and other progressive neurological disorders, are characterized by the gradual loss of neuronal structure and function and are associated with multiple interrelated mechanisms, including oxidative stress, neuroinflammation, mitochondrial dysfunction, abnormal protein aggregation, cholinergic impairment, and neuronal apoptosis. Because these pathological processes are multifactorial, phytochemicals have attracted considerable interest as potential neuroprotective agents due to their ability to act on multiple molecular targets simultaneously. Several classes of plant-derived compounds, particularly flavonoids, phenolic acids, polyphenols, alkaloids, and terpenoids, have demonstrated antioxidant, anti-inflammatory, anti-apoptotic, cholinesterase-inhibitory, and mitochondrial-protective properties, which may contribute to the preservation of neuronal function [1,6].

Oxidative stress and inflammation are central contributors to cellular injury and neuronal dysfunction, making antioxidant and anti-inflammatory interventions important therapeutic strategies. Conventional chemical approaches, although effective, are frequently constrained by toxicity, dose-dependent adverse effects, and limited activity against the multiple interconnected mechanisms underlying chronic disease. Non-steroidal anti-inflammatory drugs and selective COX-2 inhibitors can produce gastrointestinal, cardiovascular, and renal complications, particularly with prolonged use or in susceptible individuals [7,8]. Similarly, conventional acetylcholinesterase (AChE) inhibitors such as donepezil, rivastigmine, and galantamine provide symptomatic cholinergic benefits but may produce gastrointestinal and other cholinergic adverse effects, while their ability to modify the underlying progression of neurodegenerative disease remains limited [9]. These limitations support the search for safer and multifunctional agents capable of simultaneously addressing oxidative, inflammatory, and cholinergic components of neuronal injury.

Plant-derived phytochemicals may help address this therapeutic gap because individual compounds and complex extracts can exert multiple biological effects through complementary mechanisms. Phenolics and flavonoids can donate electrons or hydrogen atoms to reactive species, while terpenoids, sterols and other metabolites may influence inflammatory signalling, enzyme activity and cellular survival pathways. The importance of such multitarget activity is particularly relevant to neurotoxicity, where oxidative stress and inflammatory signalling interact to promote mitochondrial dysfunction and cell death. The available literature has established individual antioxidant, anti-inflammatory, anticonvulsant properties of *E. agallocha*, but an integrated evaluation linking phytochemical composition and neuroprotective activities needs to be addressed. Therefore, the present study aimed to comprehensively investigate the phytochemical composition and multifunctional pharmacological potential of *E. agallocha* leaves, with emphasis on antioxidant, anti-inflammatory, COX-2 inhibitory, AChE inhibitory, cyto-compatibility and TMT-associated cellular protection, followed by LC–MS-based metabolite profiling and AChE-targeted molecular docking to provide preliminary computational insights into the potential interaction of selected phytochemicals with AChE.

## 2. Results and Discussion

*Excoecaria agallocha* L. is an ecologically important mangrove belonging to the family *Euphorbiaceae* and is widely distributed throughout tropical and subtropical coastal regions of Asia, Australia, East Africa and the Pacific. The species has attracted considerable pharmacological interest because different plant parts, particularly leaves, stems, bark and roots, contain chemically diverse secondary metabolites, including diterpenoids, triterpenoids, flavonoids, sterols, phenolic compounds and other specialized metabolites. Traditional applications of *E. agallocha* include the management of rheumatism, epilepsy, paralysis, ulcers and other inflammatory or neurological conditions. A comprehensive phytochemical review reported numerous structurally diverse constituents and summarized antioxidant, anti-inflammatory, anticonvulsant, anticancer and other pharmacological activities of the species [10].

More recently, the pharmacological importance of *E. agallocha* has been reinforced by systematic and comprehensive reviews. Uddin et al. [3] emphasized the presence of diterpenoids, triterpenoids, flavonoids, polyphenols, sterols and other constituents and highlighted antioxidant, anti-inflammatory, cytotoxic and neurological activities. A recent review by Richi et al. [4] reported at least 235 phytoconstituents from different parts of *E. agallocha*, emphasizing the remarkable chemical diversity of the species and its broad pharmacological potential. Against this background, the present study investigated the biological properties of *E. agallocha* leaves through complementary phytochemical, antioxidant, anti-inflammatory, cytotoxic, neuroprotective and anti-acetylcholinesterase assays.

### 2.1. Phytochemical Screening and Quantitative Phenolic and Flavonoid Contents

The preliminary phytochemical investigation demonstrated a distinct solvent-dependent distribution of constituents in *E. agallocha* leaves. The petroleum ether extract showed the presence of only phytosterols, whereas the methanol and aqueous extract showed the presence of phenolic compounds, flavonoids, and terpenoids. The detection of phytosterols in the petroleum ether fraction is chemically reasonable because petroleum ether preferentially extracts non-polar constituents, including sterol and lipid-associated molecules. The result is also consistent with the established phytochemical profile of *E. agallocha*. Mondal et al. [10] reported sterols, triterpenoids and numerous diterpenoids from different parts of the species, while the more recent review by Richi et al. [4] confirmed the broad occurrence of sterol and terpenoid chemistry in the plant.

The detection of phenolic compounds, flavonoids, and terpenoids in the aqueous extract is particularly important in relation to the subsequent antioxidant and anti-inflammatory experiments. Phenolic compounds possess hydroxyl groups capable of donating hydrogen atoms or electrons to reactive species, whereas the flavonoids and terpenoid constituents exhibit antioxidant, anti-inflammatory and membrane-modulating properties.

The quantitative analysis further demonstrated that the water extract contained higher total phenolic and flavonoid contents than the methanol extract. Total phenolic contents were reported as 562.43 µg/g gallic acid equivalents for the methanol extract, and 717.81 µg/g for the water extract, whereas total flavonoid contents were 334.00 and 485.16 µg/g quercetin equivalents, respectively (Table 1). Thus, the aqueous extract contained approximately 27.7% more total phenolic material and approximately 45.2% more flavonoid-equivalent material than the methanolic extract.

This finding is noteworthy because earlier work has demonstrated that the antioxidant activity of *E. agallocha* leaves is closely associated with phenolic constituents. Masuda et al. [11], in a comparative investigation of leaves from 39 seashore plants, found that *E. agallocha* was among the plants exhibiting particularly strong antioxidant activity. Their subsequent chromatographic investigation identified ellagic acid as an important antioxidant constituent.

Deepa et al. [12] similarly reported significant phenolic content and antioxidant activity in *E. agallocha* leaf fractions, although the ethyl acetate fraction was the most active fraction in their experimental system. Their findings demonstrated that the phenolic-rich ethyl acetate fraction showed strong antioxidant and reducing properties as well as inhibition of protein denaturation. The difference between their solvent ranking and the present study is scientifically meaningful rather than contradictory. It indicates that the relative abundance of bioactive molecules is strongly dependent on extraction solvent, geographical origin, environmental conditions, plant maturity, drying procedure and extraction methodology [13].

The present quantitative results therefore provide an important chemical basis for interpreting the subsequent biological assays. In particular, the greater phenolic and flavonoid content of the aqueous extract corresponds with its superior performance in both DPPH and FRAP assays, suggesting that polar antioxidant constituents contribute substantially to its biological activity.

### 2.2. DPPH Radical-Scavenging Activity

The DPPH assay demonstrated a clear concentration-dependent increase in radical-scavenging activity for all *E. agallocha* leaf extracts. At 50 µg/mL, chloroform, ethyl acetate, methanol and water extracts produced 10.20 ± 1.46%, 22.21 ± 1.60%, 28.24 ± 1.24% and 35.43 ± 0.99% inhibition, respectively. At 100 µg/mL, the corresponding activities increased to 19.78%, 30.77%, 40.01% and 47.74%, while at 250 µg/mL, they increased further to 51.42%, 60.09%, 73.95% and 78.13%, respectively (Table 2).

Ascorbic acid produced 90.65% inhibition at 250 µg/mL. The concentration-dependent behaviour indicates that the radical-scavenging response was not random but increased progressively with increasing extract concentration. This is characteristic of an extract containing several molecules capable of donating hydrogen atoms or electrons to stabilize DPPH radicals. Brand-Williams et al. [14] established DPPH as a useful method for assessing radical-scavenging capacity and demonstrated that antioxidant molecules differ in both reaction rate and radical-reduction efficiency. The IC_50_ values provide a more informative comparison than the percentage inhibition at a single concentration. The IC_50_ values were 77.53 µg/mL for ascorbic acid, 249.64 µg/mL for chloroform extract, 199.31 µg/mL for ethyl acetate extract, 146.01 µg/mL for methanol extract and 115.49 µg/mL for water extract. Thus, among the *E. agallocha* fractions, the water extract was the most potent DPPH scavenger, followed by methanol, ethyl acetate and chloroform extracts.

The aqueous extract required approximately 115.49 µg/mL to achieve 50% radical inhibition, whereas the chloroform extract required approximately 249.64 µg/mL. Consequently, the aqueous fraction was approximately 2.16 times more potent than the chloroform fraction based on IC_50_. The stronger activity of the water extract corresponds closely with its higher total phenolic and flavonoid contents. This finding agrees with Masuda et al. [11], who reported remarkably strong antioxidant activity of *E. agallocha* leaf methanolic extract and identified ellagic acid as an important antioxidant constituent. Poorna et al. [15] also reported significant DPPH radical-scavenging and DNA-protective activity of *E. agallocha* leaf extract, further supporting the antioxidant potential of the species. The older literature summarized by Mondal et al. [10] similarly indicates that polar fractions of *E. agallocha* can demonstrate substantial radical-scavenging activity. These values are higher than those reported in some previous studies. The IC_50_ value of 77.53 μg/mL for ascorbic acid in the DPPH assay in comparatively higher than those reported in previous studies since these values are dependent on experimental conditions, including the concentration of the radical or substrate, reagent and enzyme concentrations, incubation time, solvent composition, temperature, pH, concentration range tested, and the mathematical model used for IC_50_ estimation. Therefore, direct numerical comparison with published values should be made only when assay conditions are comparable.

### 2.3. Ferric-Reducing Antioxidant Power

The FRAP assay demonstrated a concentration-dependent increase in reducing capacity for all *E. agallocha* leaf extracts. The absorbance of the chloroform extract increased from 0.0894 ± 0.0047 at 50 µg/mL to 0.3676 ± 0.0057 at 250 µg/mL. Similarly, the absorbance of the ethyl acetate extract increased from 0.1181 ± 0.0048 to 0.4817 ± 0.0212, while the methanol extract increased from 0.2120 ± 0.0136 to 0.8002 ± 0.0066. The water extract showed an increase from 0.2319 ± 0.0102 to 0.8947 ± 0.0234 over the same concentration range. Ascorbic acid exhibited the highest absorbance, increasing from 0.2883 ± 0.0048 at 50 µg/mL to 1.1335 ± 0.0062 at 250 µg/mL (Table 3).

The ranking of the extracts was therefore water > methanol > ethyl acetate > chloroform. This ranking is almost identical to that obtained in the DPPH assay. Such agreement between two mechanistically different antioxidant assays is particularly important because DPPH measures radical-scavenging capacity, whereas FRAP reflects electron-transfer/reducing capacity. Benzie and Strain [16] established FRAP as a measure of reducing antioxidant potential based on ferric-to-ferrous reduction and formation of a coloured ferrous complex.

The strong FRAP activity of the water extract therefore reinforces the conclusion obtained from DPPH. Importantly, the aqueous extract had both the highest total phenolic content and highest total flavonoid content, suggesting that polar phenolic constituents may be major contributors to its reducing capacity. Deepa et al. [12] also reported strong reducing activity among *E. agallocha* leaf fractions, although the ethyl acetate fraction was superior in their study. This difference further emphasizes that solvent extraction changes the chemical composition of plant fractions.

### 2.4. Anti-Inflammatory Activity Based on Protein Denaturation

The protein-denaturation assay demonstrated concentration-dependent inhibition by all four *E. agallocha* leaf extracts. Aspirin was used as the reference standard. At 50 µg/mL, aspirin produced 36.65 ± 1.38% inhibition, whereas chloroform, ethyl acetate, methanol and water extracts produced 16.53 ± 0.94%, 21.04 ± 0.78%, 31.62 ± 1.24% and 23.76 ± 1.15%, respectively. At 250 µg/mL, the activities increased to 58.65 ± 0.52%, 62.83 ± 0.97%, 75.06 ± 0.83% and 71.03 ± 1.09%, respectively, while aspirin reached 89.77 ± 0.75% (Table 4).

The calculated IC_50_ values were 96.88 µg/mL for aspirin, 209.25 µg/mL for chloroform, 189.88 µg/mL for ethyl acetate, 138.89 µg/mL for methanol, and 158.44 µg/mL for water extract. Thus, the methanol extract showed the greatest protein-denaturation inhibitory activity among the plant extracts, followed by water, ethyl acetate and chloroform extracts.

The strong activity of the methanol extract is interesting because the water extract was superior in antioxidant and COX-2 assays. This demonstrates that the biological activity of an extract cannot be predicted solely from its total phenolic content. Different solvent systems may enrich different subsets of phenolic, terpenoid, sterol, and other compounds, and individual assays may respond preferentially to different structural classes. The present findings are consistent with Deepa et al. [12], who reported significant inhibition of Bovine Serum Albumin (BSA) denaturation by *E. agallocha* leaf extracts. In their investigation, the ethyl acetate fraction produced 85.38% inhibition at 100 µg/mL. Their study therefore independently confirms that *E. agallocha* leaf constituents possess protein-stabilizing activity.

More recent evidence provides additional mechanistic support. Purushothaman et al. [17] isolated n-hexadecanoic acid from *E. agallocha* and demonstrated dose-dependent protein-denaturation inhibition, membrane stabilization and COX-1/COX-2 inhibitory effects, together with anti-inflammatory activity in a carrageenan-induced paw-edema model. This study is particularly relevant because it demonstrates that the anti-inflammatory effects of *E. agallocha* can be associated with individual constituents rather than only crude extracts. Yin et al. [18] further demonstrated that several *E. agallocha* diterpenoids suppressed NF-κB and AP-1 activation and reduced TNF-α and IL-6 expression in LPS-stimulated macrophages. Thus, the protein-denaturation results obtained in the present investigation are supported by both biochemical and molecular evidence from previous studies.

### 2.5. COX-2 Inhibitory Activity

The COX-2 inhibition assay demonstrated particularly strong activity of the aqueous *E. agallocha* leaf extract. At 20 µg/mL, the standard drug celecoxib produced 43.55 ± 1.38% inhibition compared with 15.36 ± 0.93% for chloroform, 10.38 ± 0.97% for ethyl acetate, 33.71 ± 1.23% for methanol, and 37.37 ± 0.13% for water extract. At 100 µg/mL, inhibition increased to 65.43 ± 1.89%, 53.84 ± 0.51%, 81.67 ± 0.95% and 84.68 ± 1.11% for chloroform, ethyl acetate, methanol and water extracts, respectively, compared with 90.45 ± 0.75% for celecoxib. The IC_50_ values were 34.94 µg/mL for celecoxib, 80.40 µg/mL for chloroform, 97.23 µg/mL for ethyl acetate, 45.39 µg/mL for methanol and 39.77 µg/mL for water extract (Table 5).

The aqueous extract therefore exhibited the strongest COX-2 inhibitory activity among the plant fractions. Remarkably, its IC_50_ was relatively close to that of celecoxib under the experimental conditions. This is particularly important because COX-2 is an inducible enzyme involved in prostaglandin synthesis during inflammation. Celecoxib is a selective COX-2 inhibitor, and therefore its use as the reference standard provides a pharmacologically relevant benchmark for the present assay. Nevertheless, similarity between crude-extract and celecoxib IC_50_ values should not be interpreted as equivalent pharmacological potency in vivo. Celecoxib is a purified compound with a defined molecular target, whereas the aqueous extract contains numerous compounds with potentially different affinities and mechanisms.

The strong COX-2 activity of the aqueous fraction corresponds with its strong antioxidant activity. Oxidative stress and inflammatory signalling are closely interconnected, and phenolic compounds may influence inflammatory pathways through redox regulation as well as direct modulation of inflammatory enzymes. The findings of Yin et al. [18], who demonstrated suppression of NF-κB, AP-1, TNF-α and IL-6 by *E. agallocha* diterpenoids, provide an additional explanation for the broader anti-inflammatory profile of the species.

### 2.6. Cytotoxicity on L929 Normal Fibroblast Cells

The cytotoxic effects of the methanol extract (ME) and water extract (WE) of *E. agallocha* leaves were evaluated in L929 fibroblast cells across concentrations of 50–250 µg/mL. Both extracts produced a concentration-dependent reduction in cell viability. ME reduced cell viability from 88.30 ± 1.54% at 50 µg/mL to 19.69 ± 1.44% at 250 µg/mL, whereas WE reduced viability from 93.94 ± 0.82% to 23.79 ± 0.82% over the same concentration range (Table 6).

**Figure 1:**
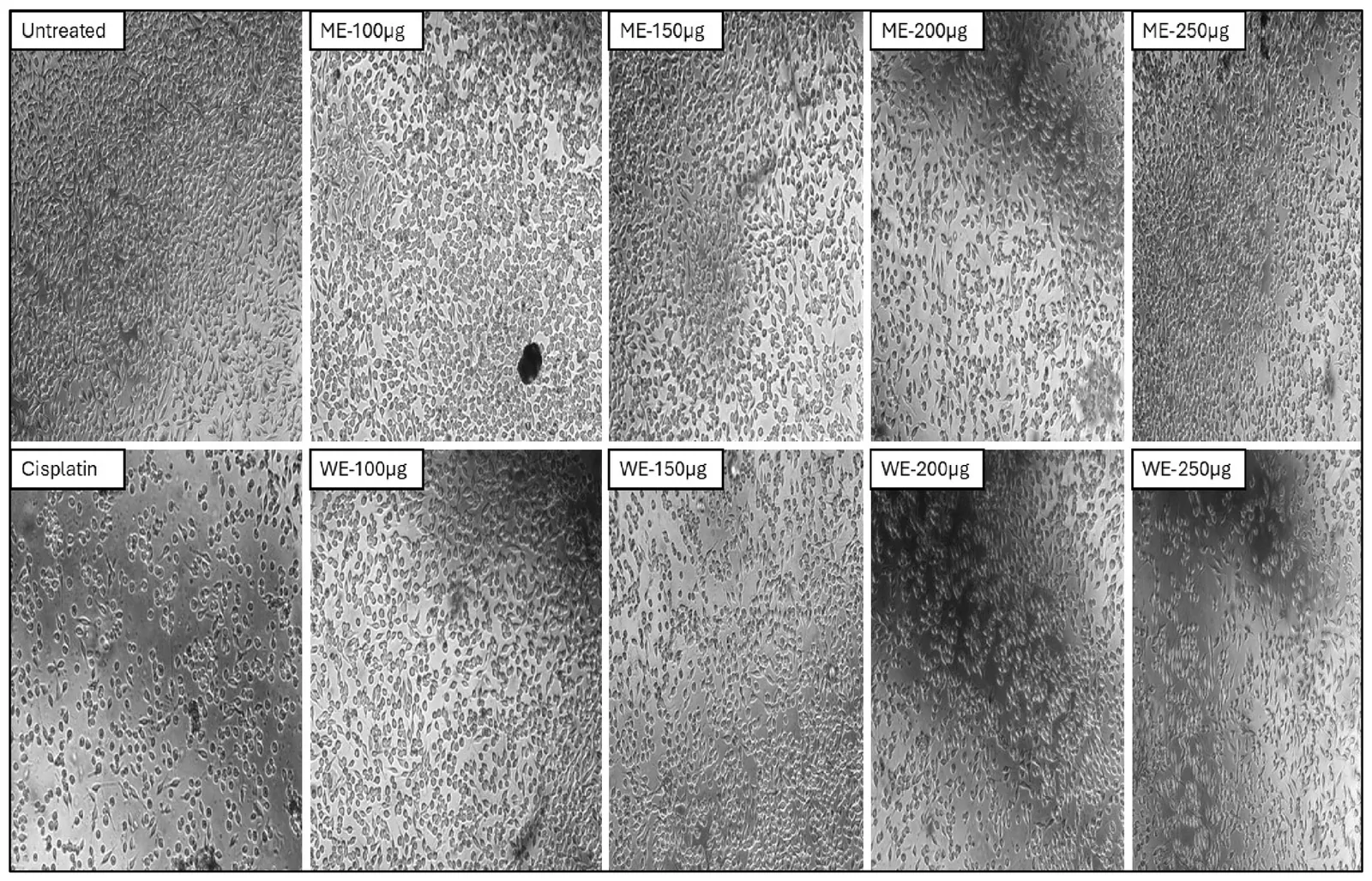
Morphological effects of test samples on L929 cell line.

The morphological effects of *E. agallocha* methanol and water extract on L929 cell line are depicted in Figure 1.

**Figure 2:**
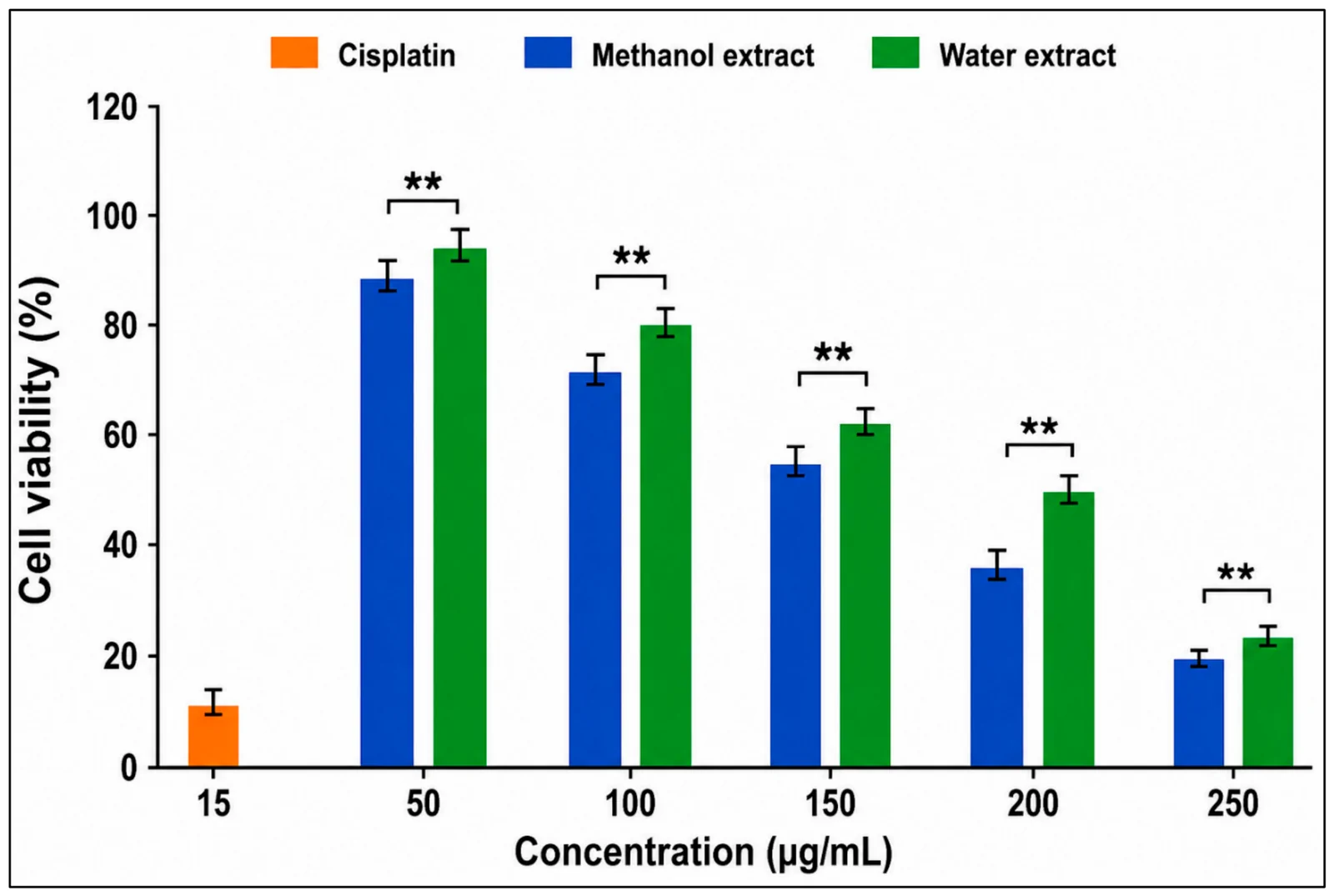
Cytotoxic effects of *E. agallocha* leaf methanol and water extracts on L929 fibroblast cells. Data are presented as mean ± SEM (*n* = 3). Statistical significance between ME and WE at corresponding concentrations was assessed using two-way ANOVA, with extract type and concentration as the two factors, followed by Tukey’s HSD multiple-comparison test. ** *p* < 0.01 for ME versus WE comparisons at the corresponding concentrations. Cisplatin (15 µg/mL) was included as a reference cytotoxic agent.

Two-way ANOVA demonstrated significant effects of treatment and concentration, with a significant treatment × concentration interaction (*p* < 0.001). Post-hoc comparisons showed that cell viability differed significantly between ME and WE at each tested concentration. Overall, the water extract maintained significantly higher cell viability than the methanol extract, particularly at the lower and intermediate concentrations, indicating comparatively lower cytotoxicity toward normal L929 fibroblasts. The calculated IC_50_ values further supported this finding, with WE showing a higher IC_50_ (184.79 µg/mL) than ME (160.50 µg/mL). Cisplatin, included as the positive cytotoxic control, produced markedly lower L929 cell viability (11.79 ± 1.74%) at 15 µg/mL (Figure 2).

The cytotoxicity findings become particularly relevant when considered together with the COX-2, AChE and neuroprotective data. The aqueous extract showed strong pharmacological activity at concentrations below its L929 IC_50_, suggesting that the biologically active concentration range may precede substantial cytotoxicity toward normal fibroblasts. This observation requires formal selectivity-index analysis in future work.

Earlier research has demonstrated that *E. agallocha* extracts can exert selective biological effects against cancer cells. For example, a recent study by Sultana et al., 2022, reported cytotoxic activity of *E. agallocha* leaf extract against cervical cancer cells (SiHa HPV 16+) while investigating effects on normal cells, supporting the concept that biological effects may differ according to cell type [19]. However, normal-cell cytotoxicity studies should be expanded using additional normal human cell types before any safety conclusion is made. The present L929 data provide useful preliminary evidence but are not sufficient for systemic toxicological evaluation.

### 2.7. Neuroprotective Activity Against TMT-Induced SK-N-SH Cell Injury

**Figure 3:**
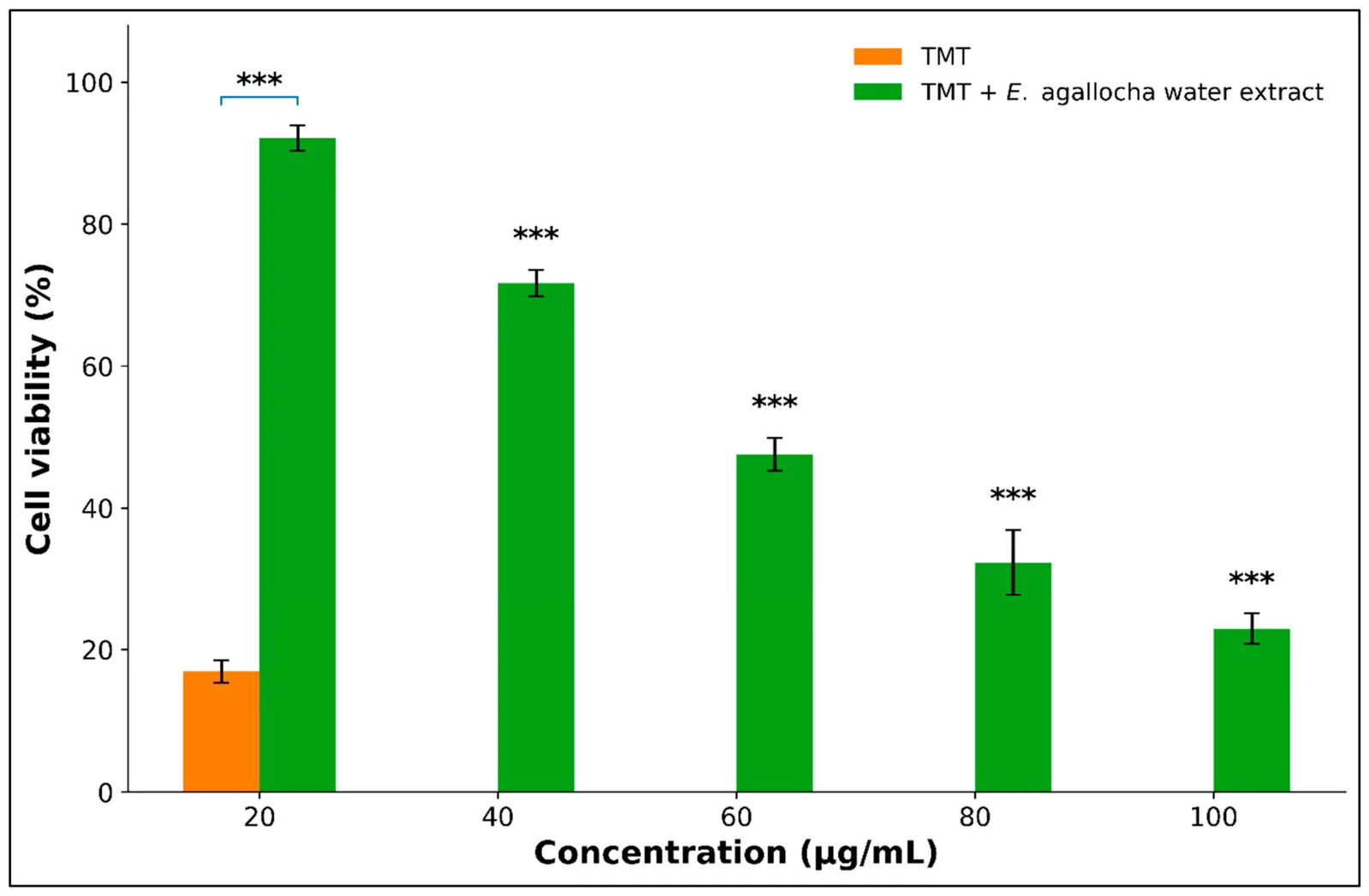
Neuroprotective effect of *E. agallocha* water extract against TMT-induced SK-N-SH cell injury. Data are presented as mean ± SEM (*n* = 3). Statistical significance was assessed using one-way ANOVA followed by Dunnett’s multiple-comparison test, with TMT alone as the control. *** *p* < 0.001 versus TMT control.

The neuroprotective experiment is one of the most important components of the present study. The *E. agallocha* leaf water extract was administered 1 h before TMT exposure, thereby testing a pre-treatment protective paradigm rather than a post-injury therapeutic paradigm. The TMT + water-extract group demonstrated 92.14 ± 1.77% viability at 20 µg/mL, 71.68 ± 1.88% at 40 µg/mL, 47.56 ± 2.32% at 60 µg/mL, 32.30 ± 4.58% at 80 µg/mL and 22.92 ± 2.16% at 100 µg/mL. In contrast, the Neurotoxicant (TMT) alone at 20 µg/mL produced 16.92 ± 1.55% viability (Table 7). Compared with TMT alone (16.92 ± 1.55%), the extract-treated groups showed significantly higher cell viability at all tested concentrations (*p* < 0.001), indicating a marked protective effect against TMT-induced neurotoxicity (Figure 3).

The difference at 20 µg/mL is significant. Pre-treatment with the water extract increased viability from 16.92% in the TMT-only group to 92.14%, representing an absolute improvement of approximately 75%. This indicates substantial protection against TMT-associated cellular injury under the present experimental conditions. The concentration-dependent decline in viability in the TMT + water-extract groups was also observed. The strongest apparent protection was observed at 20 µg/mL, whereas cell viability progressively decreased at higher extract concentrations. However, since extract-only controls were not included at each concentration, the observed decline cannot be clearly attributed to the extract itself.

TMT is a well-established neurotoxicant capable of inducing oxidative stress, mitochondrial damage, inflammatory signaling, and cell death. Song et al. [20] demonstrated that TMT exposure in human SH-SY5Y neuroblastoma cells increased pro-inflammatory cytokines and NF-κB signalling, while reducing antioxidant enzymes such as catalase and GPx-1. They further showed that antioxidants and COX-2/LOX inhibitors, including celecoxib, attenuated TMT-induced toxicity. Currently a mechanistic hypothesis rather than a demonstrated pathway, because ROS, mitochondrial membrane potential, apoptosis proteins and inflammatory markers were not measured.

Evidence from *E. agallocha* itself strengthens this hypothesis. Shelar et al. [21] demonstrated significant anticonvulsant effects of *E. agallocha* leaf ethanolic extract in maximal-electroshock and lithium-pilocarpine models, with dose-dependent effects comparable with standard anticonvulsant drugs at higher doses. More recently, Li et al. [5] isolated structurally diverse diterpenoids from *E. agallocha* and demonstrated anti-neuroinflammatory effects involving macrophage polarization and inflammasome-associated pathways. These findings are highly relevant because neuroinflammation is increasingly recognized as an important contributor to neurodegenerative and neuronal-injury processes. Thus, the current TMT experiment provides a valuable bridge between previous evidence for antioxidant and anticonvulsant properties and newer evidence for anti-neuroinflammatory activity.

### 2.8. Morphological Evidence of Neuroprotection on TMT-Challenged SK-N-SH Cells

**Figure 4:**
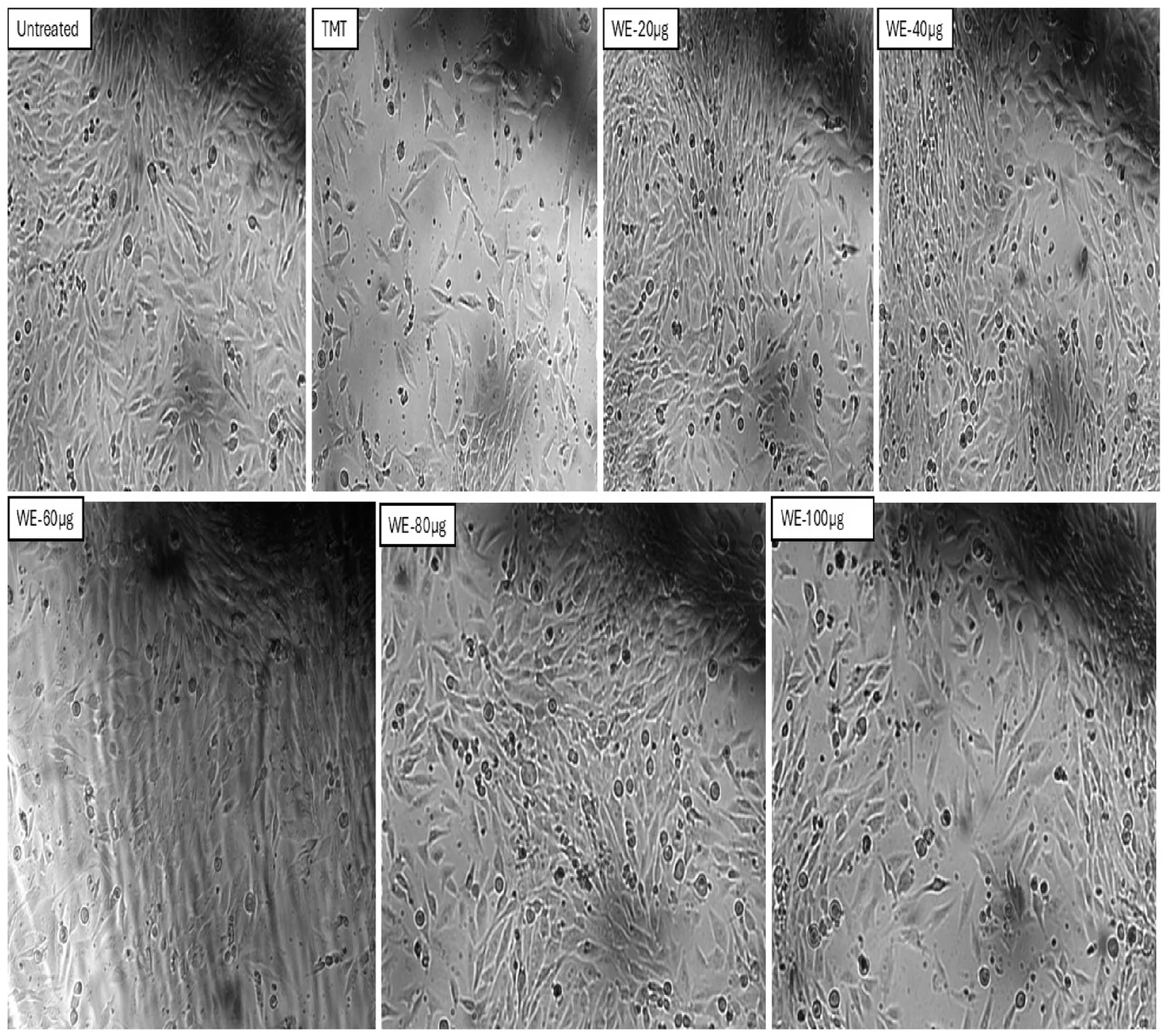
Morphological effects of test samples on neuro cell line.

The morphological images provide visual support for the quantitative cell-viability findings. The untreated cells showed relatively preserved morphology, whereas TMT-treated cells exhibited marked morphological alterations. The water-extract-pre-treated groups showed comparatively better cellular appearance, particularly at the lower concentration, consistent with the greater viability observed quantitatively (Figure 4).

Morphological assessment provides complementary information to viability assays, as changes in cell shape, adherence, and nuclear morphology can help distinguish cellular injury and apoptotic changes that may not be fully captured by metabolic viability measurements alone. Therefore, to further characterize the nature of TMT-induced cellular injury and evaluate the protective effect of *E. agallocha*, AO/EB dual fluorescence staining was performed in SK-N-SH cells. This approach enabled qualitative identification of viable, apoptotic, and severely damaged/necrotic cells based on their differential fluorescence patterns and nuclear morphology. The findings from the AO/EB staining are presented below.

### 2.9. Anti-Apoptotic Effect of Water Extract Against TMT-Induced Neurotoxicity in SK-N-SH Cells

The protective effect of the *E. agallocha* water extract against TMT-induced cellular injury was evaluated using AO/EB dual fluorescence staining. Untreated SK-N-SH cells predominantly exhibited bright green fluorescence with intact cellular and nuclear morphology, indicating viable cells with preserved membrane integrity. In contrast, exposure to TMT (20 µg/mL) resulted in a marked increase in yellow, orange, and orange-red fluorescent cells, accompanied by nuclear condensation, fragmentation, and altered cellular morphology. These changes are consistent with increased apoptotic and membrane-damage-associated cell death. AO/EB staining distinguishes viable cells from apoptotic and membrane-compromised cells based on differential fluorescence and nuclear morphology [22,23].

**Figure 5:**
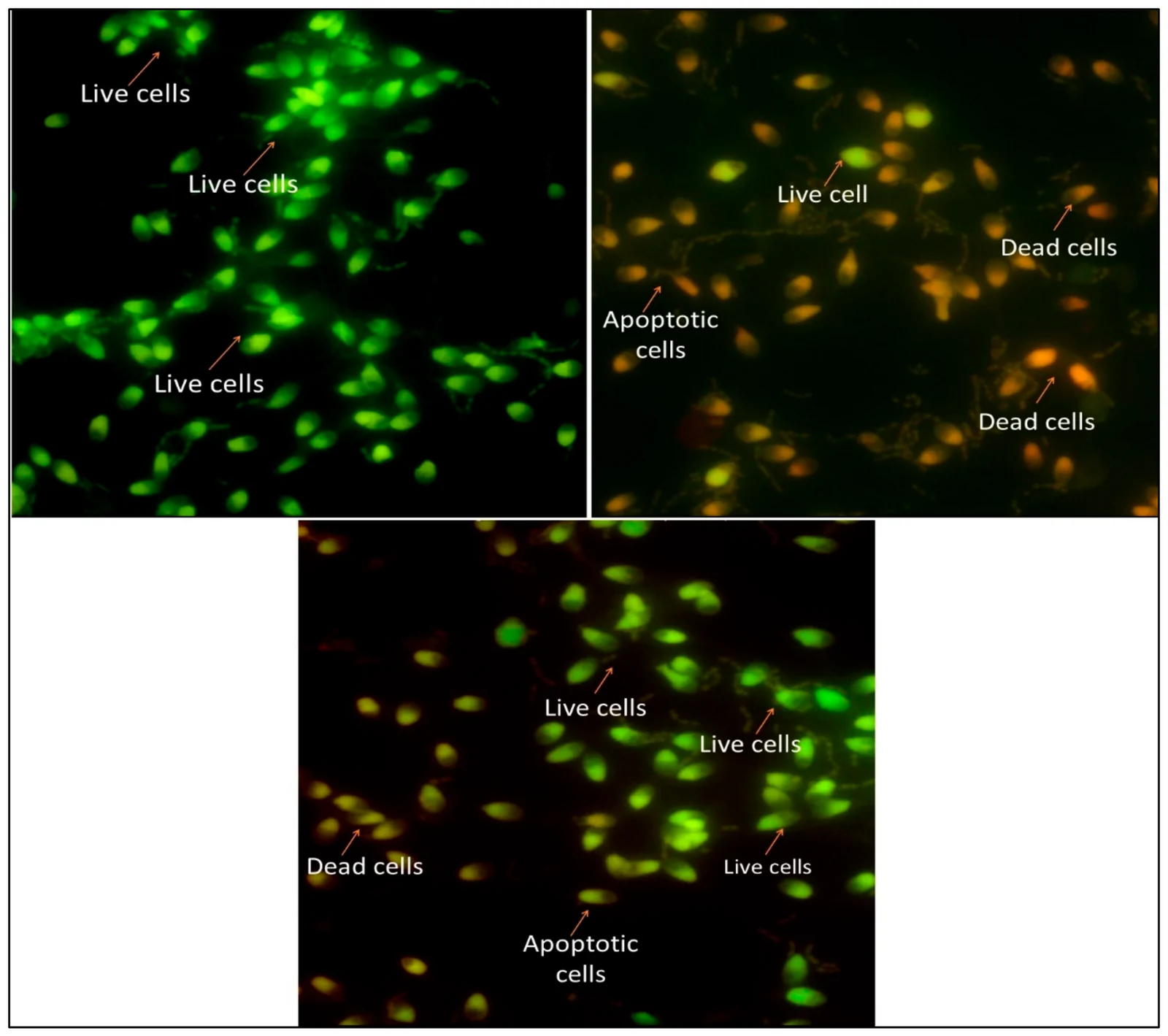
Effect of water extract on TMT-induced cellular injury in SK-N-SH cells assessed by AO/EB dual fluorescence staining.

Treatment with *E. agallocha* water extract (20 µg/mL) altered the fluorescence pattern compared with the TMT-treated group. The WE-treated cells showed a greater predominance of green fluorescence, fewer intensely orange/red fluorescent cells, and comparatively preserved cellular and nuclear morphology (Figure 5).

The shift toward a predominantly viable cellular appearance in the *E. agallocha*-treated groups suggests an improvement in cellular morphology following TMT-induced injury. Similar TMT-induced cellular changes have been reported in neuroblastoma cells and have been associated with alterations in apoptosis-related signaling pathways, including JNK, ERK, and p38, as well as changes in the expression of Bcl-2 and XIAP [24]. TMT-induced oxidative stress has also been implicated in neuronal injury and apoptosis, while antioxidant interventions have been reported to reduce TMT-associated cytotoxicity [20].

Overall, the AO/EB dual-staining findings provide qualitative morphological evidence consistent with a protective effect of *E. agallocha* against TMT-induced cellular injury in SK-N-SH cells. The predominance of viable-appearing cells and the apparent reduction in apoptosis-associated morphological changes in the treated groups suggest that the extract may mitigate TMT-induced cellular damage. However, because the AO/EB observations in the present study were based primarily on microscopic morphological assessment and were not quantitatively scored, these findings should be interpreted as supportive rather than definitive evidence of reduced apoptosis. The observed effects may be related to attenuation of oxidative stress and/or downstream apoptotic signaling; however, confirmation of the specific mechanism would require quantitative assessment of cell-death populations and molecular evaluation of apoptosis-related markers, such as caspases and Bcl-2 family proteins.

### 2.10. Anti-Acetylcholinesterase Activity

**Figure 6:**
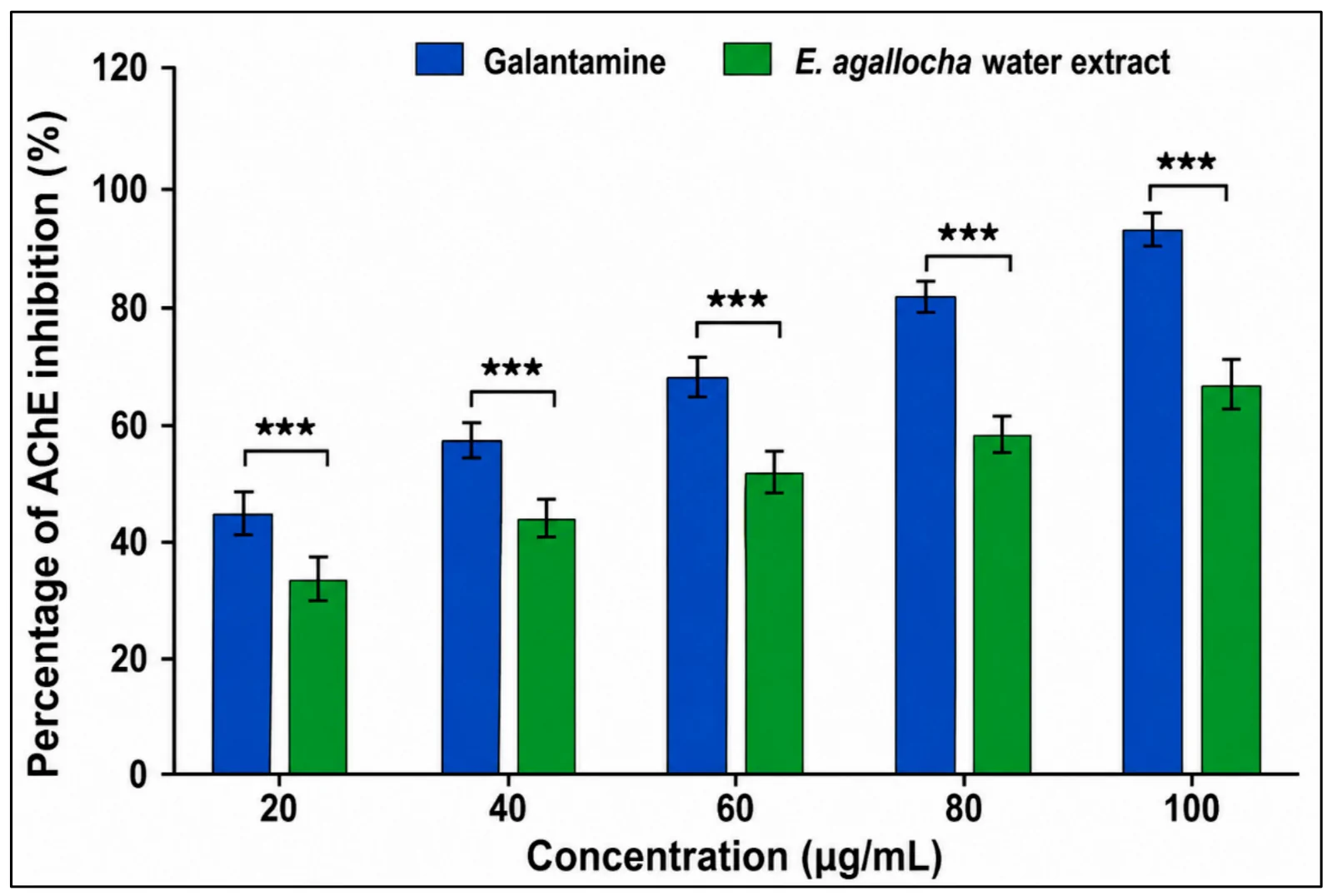
Anti-acetylcholinesterase activity of *E. agallocha* leaf water extract. Data are presented as mean ± SEM (*n* = 3). Statistical significance was assessed using two-way ANOVA, with treatment and concentration as the two factors, followed by Tukey’s HSD multiple-comparison test. *** *p* < 0.001 for comparisons between galantamine and *E. agallocha* water extract at the corresponding concentrations.

The water extract exhibited concentration-dependent AChE inhibition. The standard drug galantamine produced 44.84 ± 0.89% inhibition at 20 µg/mL, increasing to 94.88 ± 0.58% at 100 µg/mL. The *E. agallocha* water extract produced 34.63 ± 1.29%, 45.33 ± 1.41%, 52.64 ± 0.83%, 58.78 ± 0.86% and 67.13 ± 0.68% inhibition at 20, 40, 60, 80 and 100 µg/mL, respectively. The calculated IC_50_ values were 28.55 µg/mL for galantamine and 55.64 µg/mL for the water extract (Table 8 and Figure 6). The IC_50_ value for galantamine was found to be comparatively higher compared to previous studies.

The water extract therefore possessed substantial AChE inhibitory activity, although its potency was approximately two-fold lower than galantamine under the assay conditions. This is an encouraging result because crude plant extracts contain complex mixtures of constituents, whereas galantamine is a defined pharmacologically active compound. Galantamine is a reversible AChE inhibitor with additional allosteric potentiating effects at nicotinic acetylcholine receptors, making it an established pharmacological reference for cholinergic modulation [25]. The observed AChE inhibition becomes particularly interesting when integrated with the neuroprotective experiment. A single extract demonstrating antioxidant activity, COX-2 inhibition, AChE inhibition and protection against TMT-induced neurotoxicity may have multifunctional neuropharmacological potential.

The relationship is biologically plausible because oxidative stress, neuroinflammation and cholinergic dysfunction are interconnected in several neurodegenerative conditions, for instance, Alzheimer’s disease. However, the current study does not establish that AChE inhibition is responsible for the TMT-protective effect. This would require mechanistic experiments using selective AChE inhibitors, AChE knockdown or pharmacological antagonism. The finding also complements the previous anticonvulsant observations of Shelar et al. [21], who demonstrated that *E. agallocha* leaf extract reduced seizure severity in animal models.

### 2.11. LC-MS-Based Chemical Profiling

The LC-MS analysis of the *E. agallocha* leaf water extract revealed several putatively annotated metabolites belonging to diverse chemical classes. The detected compounds included an indole-related metabolite, aminocyclopropane-1-carboxylic acid, a methoxyphenol-related compound, campesterol, a methylated flavonoid, dihydrosphingosine, rosin, a catechol-related compound, a lignan glycoside, daidzein and a phenylpropanoid-related compound.

The diversity of the LC-MS profile provides a plausible chemical explanation for the multifunctional biological properties of the extract. Phenolic, flavonoid, lignan, catechol and phenylpropanoid-related compounds may contribute to antioxidant activity, whereas sterol and terpenoid-related metabolites may contribute to membrane, inflammatory and signalling effects (Table 9).

**Figure 7:**
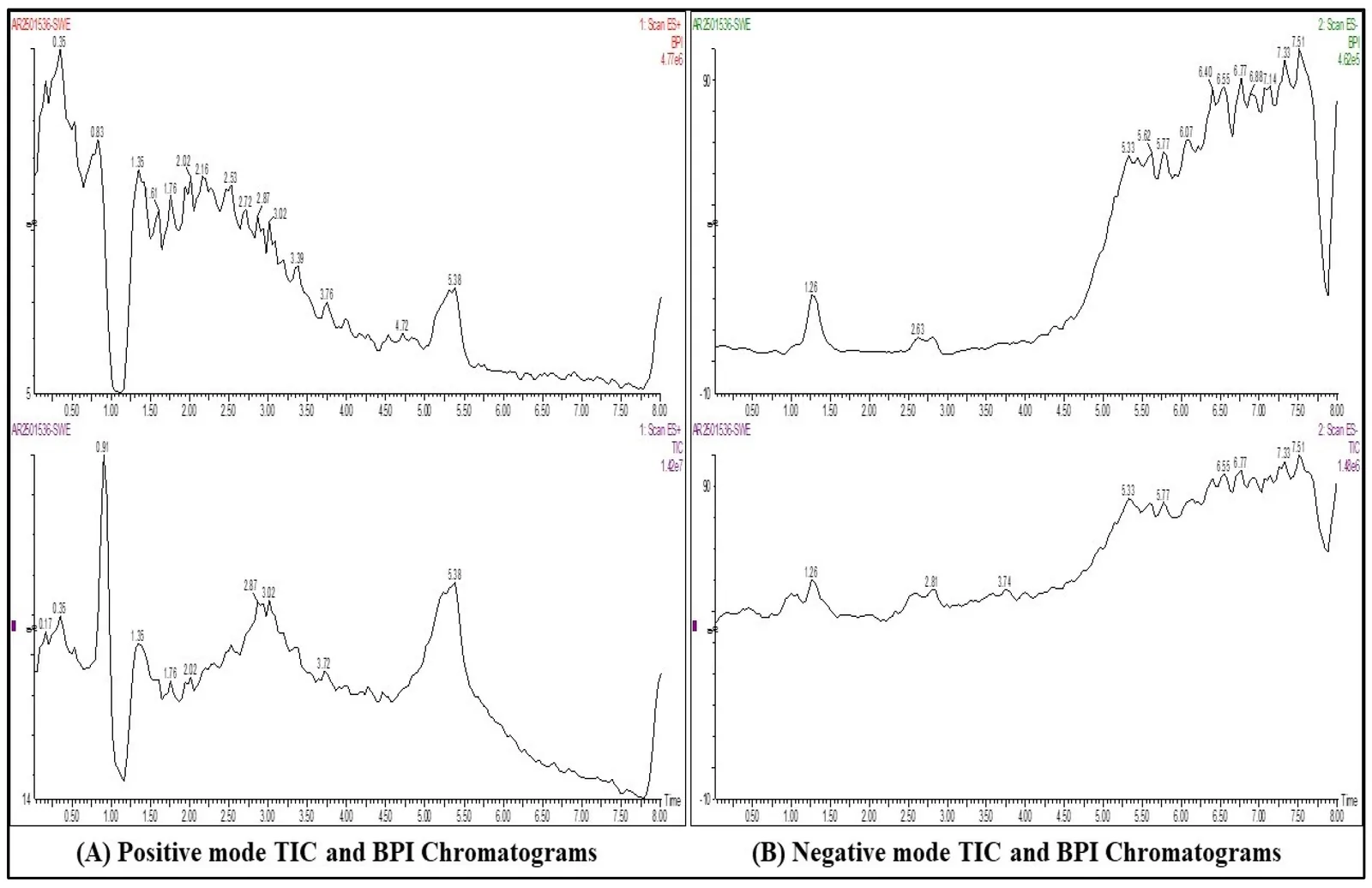
LC–MS chromatographic profiles of the *E. agallocha* water extract showing (**A**) negative-mode total ion chromatogram (TIC) and base peak intensity (BPI) chromatograms and (**B**) positive-mode TIC and BPI chromatograms.

The negative- and positive-mode TIC and BPI chromatograms showed distinct chromatographic peaks corresponding to the detected metabolite constituents (Figure 7).

The chromatographic profiles demonstrated a chemically diverse metabolite composition, supporting the putative LC-MS annotation of compounds belonging to multiple structural classes. Detailed LC–MS data, including the putatively annotated metabolites and their corresponding analytical parameters, are provided in the Supplementary File.

The putative detection of a methylated flavonoid at 1.128 min is particularly relevant because flavonoids are capable of electron transfer, hydrogen donation and interaction with oxidative and inflammatory pathways. Similarly, the putative identification of syringaresinol glycoside provides a possible explanation for phenolic and antioxidant activity. Isoflavones such as daidzein have been widely investigated for antioxidant and anti-inflammatory effects, although the presence and abundance of daidzein in the present sample should be confirmed by authentic standards and MS/MS fragmentation. The catechol-related metabolite is similarly interesting because catechol structures may participate in redox reactions and radical scavenging. Such chemistry is compatible with the strong DPPH and FRAP activities observed in the same extract. Campesterol provides additional support for the broader phytochemical observations because phytosterols were detected in the petroleum ether fraction. Mondal et al. [10] documented sterol chemistry in *E. agallocha*, demonstrating that sterol-associated constituents are part of the established phytochemical repertoire of the species.

The present LC-MS data should nevertheless be interpreted cautiously. A putative LC-MS annotation does not constitute definitive structural identification. Confirmation requires accurate mass, isotope pattern, MS/MS fragmentation and, ideally, authentic-standard comparison. This distinction is particularly important when assigning biological functions to individual compounds. The recent review by Richi et al. [4] emphasizes the remarkable chemical complexity of *E. agallocha*, with more than 235 reported phytoconstituents across multiple chemical classes. The current LC-MS profile is therefore consistent with the broader chemical diversity of the species.

The AChE inhibition results provide an additional pharmacological dimension. The aqueous extract exhibited an AChE IC_50_ of 55.64 µg, indicating meaningful inhibition compared with galantamine. The simultaneous presence of AChE inhibition, antioxidant activity and neuroprotection suggests that *E. agallocha* may possess multiple complementary neuropharmacological mechanisms. Cholinesterase inhibition can increase acetylcholine availability, while antioxidant and anti-inflammatory actions can reduce cellular stress. This is particularly relevant to neurodegenerative disease research because current therapeutic approaches increasingly emphasize multitarget strategies rather than single-target inhibition. The data demonstrate biochemical AChE inhibition and cellular protection, not disease modification. The anticonvulsant findings reported by Shelar et al. [21] provide additional support for neurological activity of the leaves. Together with the current findings, these studies suggest that *E. agallocha* deserves more detailed neuropharmacological investigation.

### 2.12. ADMET Characteristics of the Identified Compounds

The in silico ADMET analysis revealed substantial physicochemical diversity among the identified phytochemicals. The molecular weights ranged from 101.10 g/mol for 1-aminocyclopropane-1-carboxylic acid to 400.68 g/mol for campesterol. This broad molecular-weight range indicates that the compound collection contains both small polar molecules and considerably larger hydrophobic structures. 1-Aminocyclopropane-1-carboxylic acid represents the smallest molecule in the dataset, whereas campesterol represents the largest compound. The molecular-weight distribution is important because molecular size can influence passive membrane diffusion, solubility and pharmacokinetic behavior. The optimal ranges reported by commonly used drug-likeness frameworks are not absolute requirements, but they provide useful preliminary indicators during compound prioritization [26].

The hydrogen-bonding characteristics also showed considerable variation between the compounds. The relatively polar compounds, particularly 1-aminocyclopropane-1-carboxylic acid, 4-hydroxy-3-methoxyphenylglycol and daidzein, possess functional groups capable of participating in hydrogen bonding, whereas campesterol has substantially lower polarity because of its predominantly steroidal hydrocarbon framework and single hydroxyl group. These differences can influence the balance between aqueous solubility and membrane permeability. The ADMET information indicates that several compounds were predicted to have favorable gastrointestinal absorption, whereas some more hydrophobic molecules showed less favorable solubility characteristics (Table 10).

The results are particularly interesting for campesterol and 3-[(Z)-heptadec-10-enyl]benzene-1,2-diol, which showed comparatively low hydrogen-bonding/polar characteristics and less favorable solubility behavior than several of the smaller oxygenated compounds. This is chemically plausible because increasing hydrophobic surface area generally reduces aqueous solubility unless compensated by sufficient polar functionality. In contrast, the oxygen-rich flavonoid-related compounds, including 6-hydroxy-2-(4-methoxyphenyl)-4H-chromen-4-one and daidzein, possess several oxygen atoms that can contribute to hydrogen bonding and increase polarity. These properties may facilitate specific protein interactions, although excessive polarity can simultaneously influence membrane permeability and bioavailability. SwissADME and related approaches emphasize that drug-likeness should therefore be considered as a multidimensional property rather than being determined by any single parameter [26].

The overall ADMET profile suggests that the identified compounds represent chemically diverse candidates rather than a homogeneous group. This diversity is advantageous for target-based screening because compounds with different structural scaffolds can interact with different regions of a protein binding site. However, ADMET characteristics alone cannot establish biological efficacy. Instead, they provide a preliminary filter for identifying compounds that warrant additional target-based computational and experimental investigation.

### 2.13. Predicted Acute Toxicity Profile

The consolidated ProTox-II toxicity predictions demonstrated a marked difference in predicted acute oral toxicity among the investigated compounds (Table 11). The predicted LD_50_ values ranged from 225 to 5000 mg/kg, demonstrating approximately a 22-fold difference between the lowest and highest predicted LD_50_ values. 1-aminocyclopropane-1-carboxylic acid and rosin each showed a predicted LD_50_ of 5000 mg/kg and were assigned toxicity class 5.

Following duplicate checking and consolidation, nine unique compounds were included in the ADMET and ProTox-II analyses. For the subsequent AChE-targeted docking analysis, seven compounds were selected from this set based on the availability of suitable chemically defined structures for three-dimensional ligand preparation and docking. D-erythro-dihydrosphingosine and 3-[(Z)-heptadec-10-enyl] benzene-1,2-diol were not included in the docking subset, whereas the syringaresnol-related annotation and 3-(2-hydroxyphenyl) propanoate from the LC–MS profiling table were not carried forward into the computational dataset. The docking analysis was therefore intended as a focused exploratory analysis rather than an exhaustive docking evaluation of every LC–MS annotation.

The predicted toxicity of 4-hydroxy-3-methoxyphenylglycol was intermediate, with an LD_50_ of 1000 mg/kg and toxicity class 4, while campesterol had a predicted LD_50_ of 890 mg/kg and was also assigned class 4. In contrast, 6-hydroxy-2-(4-methoxyphenyl)-4H-chromen-4-one had a predicted LD_50_ of 4000 mg/kg and toxicity class 5. D-erythro-dihydrosphingosine, rosin and 3-[(Z)-heptadec-10-enyl]benzene-1,2-diol were likewise assigned class 5, with predicted LD_50_ values of 3500, 5000 and 4000 mg/kg, respectively. Daidzein showed a predicted LD_50_ of 2430 mg/kg and toxicity class 5.

These findings indicate that the compound with the strongest docking performance was not the compound with the most favorable predicted toxicity profile. This is an important observation for lead prioritization. Conversely, several compounds with weaker or intermediate docking scores were predicted to have higher LD_50_ values and class 5 toxicity. ProTox-II predictions are valuable for early safety prioritization because the platform integrates multiple computational approaches and toxicity datasets; nevertheless, they remain predictive models and should be experimentally validated before conclusions regarding safety are made [27]. The combined ADMET and toxicity findings therefore suggest that candidate selection should not rely solely on docking affinity. A compound exhibiting favorable target interaction but a comparatively unfavorable toxicity prediction may require structural optimization or additional safety evaluation, whereas a compound showing moderately favorable docking together with a more favorable predicted safety profile may represent a useful alternative lead. This integrated strategy is consistent with the broader principle of early drug discovery, where efficacy-related and developability-related characteristics must be considered simultaneously.

### 2.14. Molecular Docking Against Human Acetylcholinesterase 7E3D

AChE-targeted molecular docking was performed as a focused computational analysis of seven selected compounds from the unique LC–MS annotation set. The docking subset was defined after duplicate checking and consolidation of the LC–MS annotations and was restricted to compounds for which suitable chemically defined structures were available for three-dimensional ligand preparation. The analysis was intended to provide preliminary information on the potential interaction of selected LC–MS-annotated metabolites with human AChE rather than to represent an exhaustive docking analysis of all compounds detected by LC–MS. The predicted docking scores are therefore interpreted as relative computational estimates and not as experimental binding affinities. The analysis revealed differences in the predicted docking scores among the investigated compounds (Table 12). The strongest predicted docking score was obtained 6-hydroxy-2-(4-methoxyphenyl)-4H-chromen-4-one (−6.9 kcal/mol), campesterol and daidzein (−6.8 kcal/mol). These results represent computational predictions of relative ligand–AChE interaction and should not be interpreted as experimental measurements of binding affinity or as proof of AChE inhibition.

The difference between the best-ranked compound and the second-ranked compound was 1.4 kcal/mol, suggesting a notable difference in the docking-score ranking. However, these numerical differences should not be interpreted as experimentally equivalent differences in binding affinity because docking scoring functions are approximate and are influenced by ligand conformation, receptor preparation, solvation treatment, sampling and the mathematical form of the scoring function [28,29].

The AChE active-site gorge contains important aromatic residues, particularly Trp86, Tyr337 and Phe338, which contribute to ligand recognition and stabilization [30]. The docking pose supports its accommodation within the active-site region; however, the predicted pose represents computational evidence rather than experimental confirmation of inhibition. The second-ranked compound, 6-hydroxy-2-(4-methoxyphenyl)-4H-chromen-4-one, showed a docking score of −6.9 kcal/mol. Its rigid aromatic scaffold and oxygen-containing functional groups provide opportunities for hydrophobic, aromatic and polar interactions within the AChE gorge. Similar involvement of Trp86, Tyr337 and Phe338 has been reported in structural studies of human AChE inhibitors [30,31]. Importantly, this compound also showed a predicted LD_50_ of 4000 mg/kg and toxicity class 5, giving it a comparatively favorable integrated docking and toxicity profile.

Campesterol and daidzein both produced docking scores of −6.8 kcal/mol, although their chemical characteristics differed considerably. Campesterol possesses a rigid, hydrophobic steroidal structure that may favour interactions with nonpolar regions of the AChE gorge, consistent with the importance of hydrophobic and aromatic interactions in ligand recognition [28]. However, its relatively hydrophobic physicochemical profile and moderate solubility may limit practical development. Daidzein contains an isoflavone scaffold with hydroxyl and carbonyl groups, allowing both aromatic and hydrogen-bonding interactions. Its predicted LD_50_ of 2430 mg/kg and toxicity class 5 makes it an attractive computational candidate. The remaining compounds showed comparatively weaker docking. 4-Hydroxy-3-methoxyphenylglycol displayed a score of −5.2 kcal/mol, with substantial hydrogen-bonding potential but a smaller aromatic surface and had a predicted LD_50_ of 1000 mg/kg and toxicity class 4. Rosin showed −5.0 kcal/mol docking and a predicted LD_50_ of 5000 mg/kg, toxicity class 5. Because rosin represents a complex resinous material, its docking result should be interpreted specifically for the molecular structure evaluated rather than generalized to all rosin constituents. Chemically defined constituents should therefore be investigated further. 1-Aminocyclopropane-1-carboxylic acid exhibited the weakest docking score (−4.0 kcal/mol), but the highest predicted LD_50_ of 5000 mg/kg and toxicity class 5, illustrating that favorable predicted toxicity alone does not establish target-specific potential.

**Figure 8:**
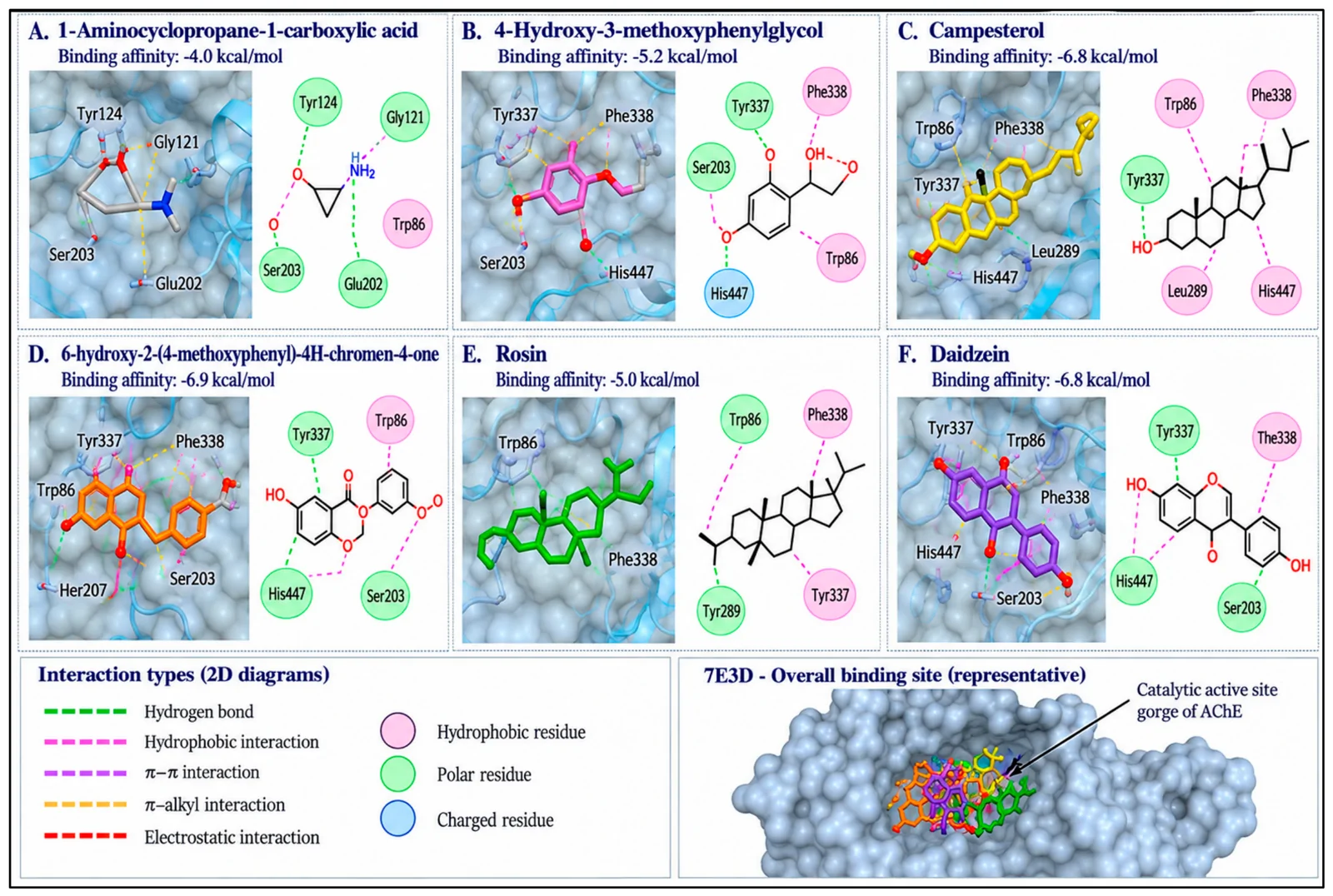
Molecular docking interactions of selected compounds within the active-site gorge of acetylcholinesterase (AChE).

The docking figures further support the computational ranking by demonstrating plausible placement of the compounds within the AChE binding gorge (Figure 8). Human AChE is structurally characterized by an aromatic-rich active-site environment containing functionally important residues such as Trp86, Tyr337, Phe338 and the catalytic Ser203/Glu334/His447 system, providing a structural basis for recognition of chemically diverse ligands.

Overall, the docking visualization showed that structurally diverse compounds could occupy the AChE binding gorge through combinations of aromatic, hydrophobic and polar interactions involving functionally important residues, including Trp86, Tyr337, Phe338, Ser203, Glu334 and His447 [28]. When docking, ADMET and toxicity findings are integrated, 6-hydroxy-2-(4-methoxyphenyl)-4H-chromen-4-one emerges as a particularly balanced candidate because of its favorable docking score and toxicity profile. Daidzein also demonstrates a promising balance of docking and predicted safety. The molecular docking results provide computational predictions of the possible interactions of selected LC–MS-annotated compounds with AChE and should not be interpreted as experimental evidence of AChE inhibition, binding affinity, pharmacological efficacy, or lead-compound validation. Experimental biochemical and pharmacological studies are required to confirm these activities.

## 3. Materials and Methods

### 3.1. Plant Collection and Authentication

Fresh, mature leaves of *Excoecaria agallocha* L. were collected from Sadashivghad, Karwar, Karnataka, India, and transported to the laboratory in clean, sterile containers to minimize contamination and deterioration. The plant was identified and authenticated according to standard botanical procedures by Dr. Haleshi C, Department of Studies in Botany, Davangere University, Davangere, Karnataka, India. The voucher specimen was assigned with deposition number HDUD533.

### 3.2. Preparation of Leaf Material

The collected leaves were carefully inspected, and extraneous materials such as soil particles, insects, damaged tissues, and other foreign matter were removed manually. The leaves were washed gently with clean water to remove adhering particulate matter and subsequently allowed to drain. The cleaned material was dried under controlled conditions, direct exposure to intense sunlight was avoided. The dried leaves were subsequently pulverized into a uniform powder using a clean laboratory grinder and stored in an airtight container under dry conditions until extraction.

### 3.3. Preparation of Solvent Extracts

The powdered leaves of *E. agallocha* were subjected to extraction in the ratio 1:10 using solvents of different polarities to obtain chemically diverse fractions with the help of a Soxhlet apparatus. Chloroform extract (CE), ethyl acetate extract (EAE), methanol extract (ME) and water extract (WE) were prepared. The use of solvents with different polarities was intended to facilitate differential recovery of non-polar, moderately polar and highly polar phytoconstituents. Solvent polarity is an important determinant of extraction efficiency and can substantially influence both the chemical profile and biological activity of plant extracts [13].

### 3.4. Preliminary Phytochemical Screening

Qualitative phytochemical screening was performed on the prepared extracts to determine the presence or absence of major classes of secondary metabolites. The screening included alkaloids, phenolic compounds, flavonoids, terpenoids, steroids, saponins, tannins, anthraquinone glycosides, phlobatannins, phytosterols, lignins, quinones, anthocyanins and coumarins. Standard qualitative chemical reactions based on characteristic colour development, precipitation or other diagnostic reactions were employed. The results were recorded as positive or negative according to the metabolites tested.

### 3.5. Determination of Total Phenolic Content

Total phenolic content of the methanolic and aqueous leaf extracts was determined using the Folin–Ciocalteu colorimetric method with slight modifications of Singleton and Rossi (1965) [32]. Briefly, 0.5 mL of diluted methanol and aqueous extracts was mixed with 2.5 mL of 10% (*v*/*v*) Folin–Ciocalteu reagent and incubated for 5 min at room temperature (25 ± 2 °C). Subsequently, 2.0 mL of 7.5% (*w*/*v*) sodium carbonate (Na_2_CO_3_) was added, mixed thoroughly, and the reaction mixture was incubated in the dark for 30 min at room temperature. The absorbance of the resulting blue-colored complex was measured at 765 nm against a reagent blank using a UV–visible spectrophotometer (LABMAN UV-vis, Tamil Nadu, India). Gallic acid (200–1000 µg/mL) was used as the reference standard gallic acid to construct the calibration curve, and the phenolic content of the extracts was calculated from the corresponding linear regression equation and expressed as mg gallic acid equivalents per gram of dry extract (µg GAE/g extract). All analyses were performed in triplicate, with appropriate reagent blanks included to correct for background absorbance.

### 3.6. Determination of Total Flavonoid Content

Total flavonoid content of the methanolic and aqueous leaf extracts was determined using the aluminium chloride (AlCl_3_) colorimetric method with quercetin as the reference standard. Briefly, 0.5 mL of appropriately diluted extract was mixed with 0.3 mL of 5% (*w*/*v*) sodium nitrite (NaNO_2_) and allowed to react for 5 min at room temperature. Subsequently, 0.3 mL of 10% (*w*/*v*) aluminium chloride (AlCl_3_) was added, followed by incubation for 6 min. Then, 2.0 mL of 1 M sodium hydroxide (NaOH) was added, and the final volume was adjusted to 5 mL with distilled water. The reaction mixture was mixed thoroughly and incubated at room temperature for 15 min, after which the absorbance was measured at 510 nm against an appropriate reagent blank using a UV–visible spectrophotometer. Quercetin solutions (200–1000 µg/mL) were used to construct the calibration curve, and the flavonoid content of the extracts was calculated from the corresponding regression equation and expressed as mg quercetin equivalents per gram of dry extract (µg QE/g extract). All measurements were performed in triplicate. The aluminium chloride method has been widely applied for comparative estimation of flavonoid-rich plant extracts [33,34].

### 3.7. DPPH Radical-Scavenging Assay

The free-radical-scavenging activity of the chloroform, ethyl acetate, methanol and water extracts was evaluated using the 2,2-diphenyl-1-picrylhydrazyl (DPPH) assay. Different concentrations of each extract (50 µg–250 µg/mL) were prepared in an appropriate solvent, and aliquots of the samples were mixed with freshly prepared DPPH radical solution (60 µmol/L in methanol). A reagent control containing DPPH solution without plant extract was maintained, while ascorbic acid was used as the reference antioxidant. The reaction mixtures were incubated under room temperature conditions for 30 min protected from excessive light, after which the decrease in DPPH absorbance was measured spectrophotometrically at approximately 517 nm. DPPH radical-scavenging activity was calculated from the reduction in absorbance relative to the control according to the standard relationship: Percentage inhibition = [(Acontrol − Asample)/Acontrol] × 100.

The concentration producing 50% radical-scavenging activity was estimated by plotting percentage inhibition against extract concentration and was expressed as the IC_50_ value. The method is based on reduction of the stable DPPH radical by hydrogen- or electron-donating antioxidants and has been extensively used for comparative evaluation of plant extracts [14].

### 3.8. Ferric-Reducing Antioxidant Power Assay

The reducing capacity of the extracts was further evaluated using the ferric-reducing antioxidant power (FRAP) assay. The FRAP reagent was freshly prepared using acetate buffer, 2,4,6-tripyridyl-s-triazine and ferric chloride according to the established FRAP principle. Appropriate concentrations of the chloroform, ethyl acetate, methanol and aqueous extracts were mixed with the FRAP reagent and incubated for the required reaction period. Reduction of ferric ions to ferrous ions resulted in formation of the blue-coloured ferrous–TPTZ complex, and absorbance was measured at approximately 593 nm. Ascorbic acid was used as the reference antioxidant. The increase in absorbance was interpreted as an indication of the ferric-reducing capacity of the extracts. The assay was performed according to the ferric-reducing principle described by Benzie and Strain [16], in which antioxidant reducing equivalents convert Fe(III) to Fe(II), producing a measurable chromogenic complex.

### 3.9. In Vitro Anti-Inflammatory Activity by Protein-Denaturation Inhibition

The anti-inflammatory potential of the *E. agallocha* leaf extracts was evaluated using inhibition of heat-induced bovine serum albumin (BSA) protein denaturation. Different concentrations of chloroform, ethyl acetate, methanol and aqueous extracts were prepared and evaluated against a protein substrate under controlled pH and temperature conditions. The assay was based on the principle that denaturation of proteins under thermal stress can be inhibited by compounds possessing stabilizing or anti-inflammatory potential. The reaction mixtures containing the test extract and protein solution were adjusted to pH 6.3 and incubated at 37 °C for 20 min and subsequently exposed to heat at 70 °C for 10 min to induce denaturation. After cooling, the absorbance of the reaction mixture was determined spectrophotometrically. Aspirin was used as the reference anti-inflammatory drug, while the corresponding reaction mixture without test extract served as the control. Percentage inhibition of protein denaturation was calculated using the absorbance values of the control and treated samples, and IC_50_ values were estimated from concentration–response curves. The assay was based on the protein-stabilization principle originally described by Mizushima and Kobayashi [35] and subsequently adapted extensively for screening natural products.

### 3.10. Anti-Inflammatory Activity by COX-2 Inhibition Assay

The cyclooxygenase-2 (COX-2) inhibitory activity of the chloroform, ethyl acetate, methanol and aqueous leaf extracts was evaluated using a commercially available COX (human) Inhibitor Screening Assay Kit (Cayman Chemical (Ann Arbor, MI, USA), Item No. 701230) containing recombinant human COX-2 enzyme. Briefly, the assay reaction mixture consisted of 160 µL of 0.1 M Tris-HCl reaction buffer (pH 8.0; containing 5 mM EDTA and 2 mM phenol), 10 µL of heme cofactor and 10 µL of recombinant human COX-2 enzyme, followed by addition of 10 µL of the test extract at different concentrations. The reaction mixtures were pre-incubated at 37 °C for 10 min. The enzymatic reaction was initiated by adding 10 µL of arachidonic acid (0.2 mM stock; 10 µM final concentrations) as the substrate and incubating for 2 min at 37 °C. The reaction was terminated by addition of 30 µL saturated stannous chloride (SnCl_2_) and incubation for a further 15 min at 37 °C, which converts the COX-derived PGH_2_ to the more stable PGF_2_α. PGF_2_α formed in the reaction was subsequently quantified using the kit-specific PGF_2_α enzyme immunoassay according to the manufacturer’s procedure, with absorbance measured at 412 nm. Celecoxib was used as the reference COX-2 inhibitor. Percentage inhibition was calculated relative to the untreated enzyme control, and concentration–response curves were generated to determine the IC_50_ values by appropriate regression analysis. All measurements were performed in triplicates as independent experiments [36].

### 3.11. Cytotoxicity Evaluation Using L929 Cells

The preliminary cytotoxicity of the methanolic and water extracts was evaluated using the L929 normal fibroblast cell line (Passage No-39). The cell line was procured from the National Centre for cell line Studies (NCCS), Pune, Maharashtra, India. Cells were maintained under standard cell-culture conditions in complete growth medium supplemented according to the requirements of the cell line and incubated in a humidified atmosphere containing approximately 5% CO_2_. Cells were seeded into appropriate multiwell culture plates and allowed to attach before treatment. Different concentrations (50 µg–250 µg/mL) of methanolic and water *E. agallocha* leaf extracts were prepared in cell-culture-compatible solvent and added to the cells. Untreated cells were maintained as the negative control, whereas cisplatin was included as the cytotoxic reference treatment. Following the 24 h of exposure period, cell viability was assessed using the MTT assay. MTT was added to the wells and viable metabolically active cells reduced the tetrazolium salt to insoluble formazan crystals. The crystals were subsequently solubilized and absorbance was measured using a microplate reader (Thermo Fisher Scientific, Waltham, MA, USA) at 570 nm. Percentage cell viability was calculated relative to untreated control cells, and concentration–response curves were used to estimate IC_50_ values. The MTT principle provides a quantitative colorimetric assessment of viable metabolically active cells and was originally established by Mosmann [37].

#### Morphological Assessment of L929 Cells

In addition to quantitative cell-viability measurements, morphological changes in L929 fibroblasts following treatment with the methanolic and aqueous extracts were examined microscopically. Untreated cells were used as the normal morphological reference, while cisplatin-treated cells represented the cytotoxic comparison. Changes in cellular density, attachment, spreading, cellular shape, membrane appearance, and the presence of rounded or detached cells were documented using an inverted phase-contrast microscope (Magnus Opto Systems, New Delhi, India). The morphological observations were interpreted together with the MTT viability data to provide complementary evidence regarding concentration-dependent cellular effects.

### 3.12. TMT-Induced Neurotoxicity and Neuroprotective Assay in SK-N-SH Cells

The neuroprotective potential of the aqueous *E. agallocha* leaf extract was evaluated using a trimethyltin (TMT)-induced neuronal injury model in the human neuroblastoma SK-N-SH cell line. The cell line was procured from the National Centre for cell line Studies (NCCS), Pune, Maharashtra, India. Cells were maintained in Eagle’s Minimum Essential Medium (EMEM) supplemented with 10% fetal bovine serum (FBS) and 1% penstrip solution at 37 °C in a humidified atmosphere containing 5% CO_2_. SK-N-SH cells were seeded and allowed to attach and stabilize before treatment. The aqueous leaf extract was prepared at concentrations ranging from 20 to 100 µg/mL and added to the cells 1 h before TMT exposure. Following pre-treatment, TMT was added at a final concentration of 20 µg/mL, and the cells were incubated for the predetermined experimental period. Cells treated with TMT alone (20 µg/mL) served as the neurotoxic control, whereas untreated cells served as the baseline cellular control. Following treatment, cellular viability was assessed using the MTT assay, and cellular morphological changes were examined using an inverted phase-contrast microscope. The experimental design therefore evaluated the ability of the aqueous *E. agallocha* extract to protect SK-N-SH cells against TMT-induced neuronal injury when administered before the neurotoxic challenge. TMT-induced neuronal injury has been associated with oxidative and inflammatory cellular responses, supporting the use of this model for evaluating the neuroprotective potential of bioactive plant extracts [38].

### 3.13. Assessment of Acetylcholinesterase Inhibitory Activity

The acetylcholinesterase (AChE) inhibitory activity of the aqueous leaf extract of *E. agallocha* was evaluated using a spectrophotometric assay based on the Ellman method [20]. Briefly, the assay mixture contained AChE enzyme from electric eel (*Electrophorus electricus*) (final concentration 0.1 U/mL) prepared in 0.1 M sodium phosphate buffer (pH 8.0). Different concentrations of the aqueous E. agallocha leaf extract were prepared in the same buffer and pre-incubated with the enzyme for 10 min at room temperature (25 ± 2 °C). The enzymatic reaction was initiated by addition of acetylthiocholine iodide (final concentration 0.075 mM) as the substrate and 5,5′-dithiobis-(2-nitrobenzoic acid) (DTNB; final concentration 0.01 mM) as the chromogenic reagent. AChE hydrolyses acetylthiocholine to generate thiocholine, which subsequently reacts with DTNB to produce the yellow-coloured 5-thio-2-nitrobenzoate (TNB) ion. The formation of TNB was monitored spectrophotometrically at 412 nm, and the increase in absorbance was recorded during the reaction. The enzyme control contained all assay components except the plant extract and represented 100% enzyme activity, whereas sample blanks containing the extract without enzyme were included to correct for any intrinsic colour or absorbance contributed by the extract. Galantamine was used as the reference AChE inhibitor under identical experimental conditions. The percentage inhibition of AChE activity was calculated relative to the enzyme control using the difference in absorbance after correction for sample blanks, and the IC_50_ value was determined by plotting the percentage inhibition against the corresponding extract concentration and fitting a concentration–response curve. The assay principle follows the classical Ellman procedure, in which enzymatically generated thiocholine reacts with DTNB to form the chromogenic TNB ion, which is quantified at 412 nm [39].

### 3.14. Attenuation of TMT-Induced Apoptosis in SK-N-SH Cells by Dual Staining

The protective effect of the *E. agallocha* extract against TMT-induced cytotoxicity was assessed in SK-N-SH human neuroblastoma cells using acridine orange/ethidium bromide (AO/EB) dual fluorescence staining. The cells were seeded in culture plates and allowed to adhere under standard culture conditions before being divided into untreated control, TMT-treated, and WE-treated groups. The untreated cells served as the negative control, while the toxicant group was exposed to TMT at 20 µg/mL. Cells in the treatment group were exposed to WE at its previously determined IC_50_ concentration under the experimental conditions. Following treatment, the cells were gently washed with phosphate-buffered saline and stained with a freshly prepared AO/EB working solution. Approximately 25 µL of the cell suspension was mixed with 1 µL of the staining solution and immediately examined under a fluorescence microscope. Cell viability and death-associated morphological changes were assessed from the fluorescence pattern and nuclear morphology. Cells showing uniform green fluorescence were considered viable, whereas yellow-green fluorescence with nuclear condensation or fragmentation was considered indicative of early apoptosis. Orange to red fluorescence with condensed or fragmented nuclei was interpreted as late apoptosis, while intense orange-red fluorescence with marked membrane compromise was considered indicative of necrotic or severely damaged cells [22,23]. Representative fluorescence images were captured for comparison among the untreated, TMT-treated, and WE-treated groups.

### 3.15. LC-MS-Based Chemical Profiling of the Aqueous Extract

The prepared *E. agallocha* leaf extract was subjected to liquid chromatography–mass spectrometry (LC–MS) for preliminary profiling and putative identification of its chemical constituents. Chromatographic separation was performed using a Waters (Milford, MA, USA) 2695 Separations Module equipped with an Accucore C18 column (50 × 4.6 mm, 5 µm). The mobile phase consisted of 10 mM ammonium acetate in water (mobile phase A) and acetonitrile (mobile phase B), with a 10 µL injection volume. The gradient was programmed from 90:10 (A:B) initially to 2:98 at 3 min, maintained until 6 min, and subsequently returned to 90:10 at 6.5 min and maintained until 8 min. Mass spectrometric detection was carried out using a Waters Micromass ZQ mass spectrometer with electrospray ionization in positive and negative modes. The instrument was operated with a capillary voltage of 2.8 kV, cone voltage of 30 V, source temperature of 140 °C, desolvation temperature of 400 °C and desolvation gas flow of 600 L/h. Chromatographic and mass-spectral data were processed using MassLynx V4.1. The LC–MS analysis generated total ion chromatograms and base-peak chromatograms in both positive and negative ionization modes, allowing detection of multiple mass spectral features across the chromatographic run. Detected compounds were evaluated based on their retention time, observed *m*/*z* values, molecular/adduct ions, and corresponding mass spectra. The LC–MS profile was therefore used for qualitative characterization of the extract and for identifying putative compounds potentially contributing to its observed biological activities [40,41].

### 3.16. In Silico ADMET, Toxicity and Molecular Docking Analysis

#### 3.16.1. ADMET and Drug-likeness Assessment of Selected Phytochemicals

The LC–MS-annotated compounds were initially compiled into a master list and manually curated to remove duplicate annotations, differences in capitalization, and repeated entries arising from detection in positive and negative ionization modes. Only unique annotations for which an unambiguous chemically defined structure could be assigned were considered for in silico analysis. The corresponding chemical structures were represented using their canonical molecular structures/SMILES and submitted individually to the SwissADME web-based platform for physicochemical, pharmacokinetic and drug-likeness assessment [26]. The analysis included molecular weight, hydrogen-bond acceptor and hydrogen-bond donor counts, gastrointestinal absorption, aqueous solubility, lipophilicity and drug-likeness-related parameters. A subset of structurally defined compounds was subsequently selected for AChE-targeted molecular docking based on the availability of an unambiguous chemical structure suitable for three-dimensional ligand preparation and computational docking. The selection workflow was therefore: LC–MS annotation → duplicate checking and consolidation → ADMET/toxicity assessment of unique compounds → selection of structurally suitable compounds for AChE docking [26].

#### 3.16.2. In Silico Toxicity Prediction

Toxicity was predicted using the ProTox-II web server. The same unique, chemically defined compounds used for the ADMET analysis were submitted individually as molecular structures to the platform. The predicted acute oral LD_50_ values (mg/kg) and corresponding toxicity classes were recorded. ProTox-II integrates molecular similarity, pharmacophore-based information, structural fragment features and machine-learning models to generate toxicity predictions and associated confidence information. The toxicity classes were interpreted according to the classification provided by the ProTox-II platform, with lower predicted LD_50_ values indicating greater predicted acute oral toxicity and higher predicted LD_50_ values indicating lower predicted acute oral toxicity [27]. The values reported in Table 11 were treated as computational predictions intended for preliminary prioritization and not as substitutes for experimental toxicological studies.

#### 3.16.3. Protein Structure and Molecular Docking

Molecular docking was performed using Auto Dock Vina version-1.5.7 specifically against human acetylcholinesterase (AChE). The three-dimensional structure of human AChE was obtained from the Protein Data Bank using PDB ID 7E3D with co-ordinates of X = 8.254730, Y = 117.363603, and Z = 56.676023). The exhaustiveness parameter was set to 8. The receptor structure was prepared by removing non-essential crystallographic molecules and retaining the protein structure required for docking. Hydrogen atoms and appropriate atomic charges were assigned before docking. The LC–MS-annotated compounds were converted into suitable three-dimensional ligand structures and prepared for docking using the selected docking workflow. PDB ID 7E3D is an X-ray crystal structure of human acetylcholinesterase (AChE) determined at 2.50 Å resolutions. The RCSB Protein Data Bank entry lists PE8 [3,6,9,12,15,18,21-heptaoxatricosane-1,23-diol] as the deposited small-molecule ligand. The present docking analysis used the AChE structure represented by PDB ID 7E3D. The Dileep et al. [31] publication was referred for structural information on human AChE; however, the tacrine-containing structure reported in that study should not be conflated with the PE8 ligand annotation of PDB ID 7E3D; and was not used as the biological reference inhibitor for the present docking study. The ligand structures were considered in their appropriate three-dimensional molecular conformations and evaluated against the AChE binding region. Molecular docking provides a computational approach for sampling possible ligand orientations and conformations within a protein binding site, followed by scoring of the generated poses. AutoDock Vina is one commonly used docking platform that combines conformational search with an empirical scoring function for ranking predicted protein–ligand complexes [42].

#### 3.16.4. Interpretation of Docking Scores and Interactions

The docking scores were used only for relative computational comparison of the predicted interactions of the selected compounds with AChE. More negative docking scores were interpreted as more favorable predicted binding within the limitations of the scoring function. These values should not be interpreted as experimentally measured binding affinities or as evidence that the docked compounds are responsible for the observed biological activities. In particular, the AChE docking results were not used to explain the experimentally observed anti-inflammatory (COX-2 inhibition) and antioxidant effect.

### 3.17. Statistical Analysis

All biochemical and cell-based were performed in triplicate, and data were expressed as mean ± SEM. For cell-based assays, including cytotoxicity (L929) and AChE inhibition assay, the effects of treatment and concentration on the respective measured outcomes were evaluated using two-way analysis of variance (ANOVA), including the treatment × concentration interaction where applicable. Following a significant ANOVA, Tukey’s HSD multiple-comparison test was performed to determine differences between treatment groups at individual concentrations. For the neuroprotective assay, the differences in cell viability among the TMT + *E. agallocha* water extract treatment groups were evaluated using one-way ANOVA, followed by Dunnett’s multiple-comparison test using the TMT group as the reference control. A *p*-value < 0.05 was considered statistically significant.

## 4. Conclusions

The present study demonstrates that the leaves of *Excoecaria agallocha* L. possess potential antioxidant, anti-inflammatory, COX-2 inhibitory, AChE inhibitory, and neuroprotective activities. Among the tested extracts, the aqueous extract exhibited good in vitro antioxidant, COX-2 inhibitory, and neuroprotective effects, which were associated with its comparatively higher phenolic and flavonoid contents. The aqueous extract also significantly inhibited AChE and protected SK-N-SH cells against TMT-induced cytotoxicity, suggesting a potential multifunctional neuroprotective effect. LC-MS profiling revealed diverse putatively identified metabolites that may contribute to the observed biological activities. Overall, these findings support *E. agallocha* leaves as a potential source of multifunctional bioactive compounds. Among the compounds evaluated through in silico analysis, 6-hydroxy-2-(4-methoxyphenyl)-4H-chromen-4-one and daidzein exhibited favorable predicted binding interactions and were classified as Class 5 in the in silico toxicity prediction analysis. These findings identify the compounds as potential constituents of interest for further investigation; however, the docking and toxicity results are computational predictions and do not establish their biological activity, safety, or mechanistic relevance. Experimental validation using isolated compounds is therefore required to determine their contribution to the observed pharmacological activities.

## Figures and Tables

**Table 1:** Phytochemical analysis of solvent extracts.

Quantitative Phytochemical Analysis		
	Methanol Extract	Aqueous Extract
Total phenolic content (µg/g, gallic acid equivalent)	562.43 µg/g	717.81 µg/g
Total flavonoid content (µg/g, quercetin equivalent)	334.00 µg/g	485.16 µg/g

**Table 2:** Antioxidant activity of different plant extracts using DPPH assay.

Concentration in µg/mL	Standard Ascorbic Acid	CE	EAE	ME	WE
50	43.23 ± 1.1988	10.20 ± 1.4605	22.21 ± 1.5993	28.24 ± 1.2423	35.43 ± 0.9858
100	56.29 ± 0.6188	19.78 ± 0.8263	30.77 ± 1.2861	40.01 ± 0.6107	47.74 ± 0.7850
150	66.03 ± 1.1150	28.52 ± 0.9718	40.14 ± 1.2341	49.68 ± 0.8547	58.64 ± 0.6136
200	78.85 ± 0.2803	38.89 ± 0.8684	49.91 ± 0.7715	62.66 ± 0.7726	65.73 ± 0.7130
250	90.65 ± 0.8140	51.42 ± 1.2440	60.09 ± 1.0393	73.95 ± 0.8322	78.13 ± 0.5460
IC_50_ in µg/mL	77.53	249.64	199.31	146.01	115.49

The results are expressed as mean ± standard error mean. CE: Chloroform extract; EAE: Ethyl acetate extract; ME: Methanol extract; WE: Water extract.

**Table 3:** Antioxidant activity of different plant extracts using FRAP assay.

Concentration in µg/mL	Standard Ascorbic Acid	CE	EAE	ME	WE
50	0.2883 ± 0.0048	0.0894 ± 0.0047	0.1181 ± 0.0048	0.2120 ± 0.0136	0.2319 ± 0.0102
100	0.4978 ± 0.0112	0.1440 ± 0.0050	0.1951 ± 0.0030	0.3379 ± 0.0069	0.4348 ± 0.0039
150	0.7853 ± 0.0055	0.2427 ± 0.0072	0.3119 ± 0.0069	0.4973 ± 0.0054	0.5297 ± 0.0045
200	0.9878 ± 0.0025	0.3051 ± 0.0087	0.3797 ± 0.0131	0.6854 ± 0.0047	0.7344 ± 0.0088
250	1.1335 ± 0.0062	0.3676 ± 0.0057	0.4817 ± 0.0212	0.8002 ± 0.0066	0.8947 ± 0.0234

The results are expressed as mean ± standard error mean. CE: Chloroform extract; EAE: Ethyl acetate extract; ME: Methanol extract; WE: Water extract.

**Table 4:** Anti-inflammatory activity of plant extracts using the protein denaturation assay.

Concentration in µg/mL	Standard Aspirin	CE	EAE	ME	WE
50	36.65 ± 1.3764	16.53 ± 0.9400	21.04 ± 0.7795	31.62 ± 1.4356	23.76 ± 1.1495
100	51.39 ± 1.3406	25.22 ± 0.6733	31.07 ± 0.9206	41.52 ± 1.5945	36.21 ± 1.1019
150	65.45 ± 0.9885	36.27 ± 0.2856	41.65 ± 1.6241	52.19 ± 0.9097	47.79 ± 0.6904
200	76.50 ± 0.8613	49.21 ± 0.4706	51.79 ± 1.1403	61.44 ± 1.4620	61.07 ± 1.4413
250	89.77 ± 0.7512	58.65 ± 0.5183	62.83 ± 0.9713	75.06 ± 0.5912	71.03 ± 1.0908
IC_50_ in µg/mL	96.88	209.25	189.88	138.89	158.44

The results are expressed as mean ± standard error mean. CE: Chloroform extract; EAE: Ethyl acetate extract; ME: Methanol extract; WE: Water extract.

**Table 5:** Anti-inflammatory activity of different solvent extracts using the COX-2 assay.

Concentration in µg/mL	Standard	CE	EAE	ME	WE
20	43.55 ± 0.5594	15.36 ± 0.9260	10.38 ± 0.9726	33.71 ± 1.2294	37.37 ± 0.1252
40	51.03 ± 0.4825	24.15 ± 0.5163	18.13 ± 0.9496	48.90 ± 0.6527	51.00 ± 0.9719
60	62.50 ± 0.9144	33.05 ± 0.8997	26.56 ± 1.1364	58.20 ± 0.5450	62.57 ± 0.8768
80	75.60 ± 0.9605	48.32 ± 1.5296	39.88 ± 0.9947	70.32 ± 0.8126	73.64 ± 1.3225
100	90.45 ± 0.5751	65.43 ± 1.8881	53.84 ± 0.5086	81.67 ± 0.9534	84.68 ± 1.1056
IC_50_ in µg/mL	34.94	80.40	97.23	45.39	39.77

The results are expressed as mean ± standard error mean. CE: Chloroform extract; EAE: Ethyl acetate extract; ME: Methanol extract; WE: Water extract.

**Table 6:** Cytotoxicity of selected plant extracts on L929 cell line.

Treatments	Concentration in µg/mL	Percentage of Cell Viability	IC_50_ in µg/mL
ME	50	88.30 ± 1.5384	160.50
	100	70.66 ± 1.7435	
	150	54.35 ± 1.8461	
	200	35.07 ± 1.2357	
	250	19.69 ± 1.4359	
WE	50	93.94 ± 0.8205	184.79
	100	80.10 ± 1.5384	
	150	61.74 ± 1.2307	
	200	49.74 ± 0.7179	
	250	23.79 ± 0.8205	
Cisplatin	15	11.79 ± 1.7435	-

The results are expressed as mean ± standard error mean. ME: Methanol extract; WE: Water extract.

**Table 7:** Neuroprotective activities of test samples on the Neuro SK-N-SH cell line.

Treatments	Concentration in µg/mL	Percentage of Cell Viability
TMT + Water extract	20	92.14 ± 1.7699
	40	71.68 ± 1.8805
	60	47.56 ± 2.3230
	80	32.30 ± 4.5751
	100	22.92 ± 2.1608
TMT	20	16.92 ± 1.5486

The results are expressed as mean ± standard error mean.

**Table 8:** AchE inhibitory activity of *E. agallocha* leaf water extract.

Treatments	Concentration in µg/mL	Percentage of Inhibition	IC_50_ in µg/mL
Standard galantamine	20	44.84 ± 0.8895	28.55
	40	57.62 ± 0.4052	
	60	68.42 ± 0.7475	
	80	82.26 ± 0.4002	
	100	94.88 ± 0.5824	
WE	20	34.63 ± 1.2881	55.64
	40	45.33 ± 1.4088	
	60	52.64 ± 0.8334	
	80	58.78 ± 0.8628	
	100	67.13 ± 0.6806	

The results are expressed as mean ± standard error mean. WE: Water extract.

**Table 9:** Tentative LC–MS annotation of molecular features detected in the *E. agallocha* leaf aqueous extract.

Ionization Mode	RT (min)	Observed *m*/*z*	Tentative Annotation	Ion/Adduct
Positive	0.869	~102.23	1-Aminocyclopropane-1-carboxylic acid *	Positive ion
Positive	1.017	~207.10	4-Hydroxy-3-methoxyphenylglycol *	[M + Na]^+^
Positive	1.091	~401.24	Campesterol *	[M + H]^+^
Positive	1.128	~291.09	6-Hydroxy-2-(4-methoxyphenyl)-4H-chromen-4-one *	[M + Na]^+^
Positive	1.572	~302.46	D-erythro-Dihydrosphingosine *	[M + H]^+^
Positive	2.682	~297.13	Rosin *	[M + H]^+^
Positive	2.904	~347.20	3-[(Z)-Heptadec-10-enyl]benzene-1,2-diol *	[M + H]^+^
Positive	3.052	—	Syringaresnol-related glycoside *	[M + H]^+^
Positive	3.089	—	Daidzein *	[M + H]^+^
Negative	0.888	—	1-Aminocyclopropane-1-carboxylic acid *	[M − H]^−^
Negative	2.479	—	3-(2-Hydroxyphenyl)propanoate *	[M − H]^−^

* Tentative annotations are based on LC–MS data; identities have not been confirmed using authentic reference standards.

**Table 10:** ADMET Properties of identified compounds in LC-MS.

Compound Name	Smilies	mf	mw	HA	HD	GI	Solubility	Lipinski
1-Aminocyclopropane-1-carboxylic acid	OC(=O)C(N)(C1)C1	C_4_H_7_NO_2_	101.10 g/mol	3	2	high	Soluble	yes
4-Hydroxy-3-MethoxyPhenylglycol	COC1=C(C=CC(=C1)C(CO)O)O	C_9_H_12_O_4_	184.19 g/mol	4	3	high	Soluble	yes
Campesterol	OC1CC2=CCC3C(CCC4(C)C(CCC34)C(C)CCC(C)C(C)C)C2(C)CC1	C_28_H_48_O	400.68 g/mol	1	1	low	Moderately Soluble	yes
6-hydroxy-2-(4-methoxyphenyl)-4H-chromen-4-one	O=C1C=C(OC2=CC=C(O)C=C12)C=3C=CC(OC)=CC3	C_16_H_12_O_4_	268.26 g/mol	4	1	high	Soluble	yes
D-erythro-Dihydrosphingosine	CCCCCCCCCCCCCCC[C@@H](O)[C@@H](N)CO	C_18_H_39_NO_2_	301.51 g/mol	3	3	high	Moderately Soluble	yes
Rosin	OCC1OC(OCC=CC=2C=CC=CC2)C(O)C(O)C1O	C_15_H_20_O_6_	296.32 g/mol	6	4	high	Soluble	yes
3-[(Z)-heptadec-10-enyl]benzene-1,2-diol	OC1=CC=CC(=C1O)CCCCCCCCCC=CCCCCCC	C_23_H_38_O_2_	346.55 g/mol	2	2	low	poorly soluble	yes
Daidzein	Oc(c3)ccc(c3)C(=C1)C(=O)c(c2)c(cc(O)c2)O1	C_15_H_10_O_4_	254.24 g/mol	4	2	high	Soluble	yes

**Table 11:** Complete consolidated ProTox-II toxicity results.

Sl. No.	Putatively Annotated Compounds	Predicted LD_50_ (mg/kg)	Predicted Toxicity Class	Overall ProTox Finding
1	1-Aminocyclopropane-1-carboxylic acid	5000	5	Highest predicted LD_50_; class 5 prediction.
2	4-Hydroxy-3-MethoxyPhenylglycol	1000	4	Intermediate predicted LD_50_; class 4 prediction.
3	Campesterol	890	4	Class 4 prediction with relatively high average similarity.
4	6-hydroxy-2-(4-methoxyphenyl)-4H-chromen-4-one	4000	5	High predicted LD_50_; class 5 prediction.
5	D-erythro-Dihydrosphingosine	3500	5	Class 5 prediction with the highest reported similarity and prediction accuracy.
6	Rosin	5000	5	Highest predicted LD_50_; class 5 prediction.
7	3-[(Z)-heptadec-10-enyl]benzene-1,2-diol	4000	5	High predicted LD_50_; class 5 prediction.
8	Daidzein	2430	5	Class 5 prediction with high average similarity and the highest reported prediction accuracy after D-erythro-Dihydrosphingosine.

**Table 12:** AChE-targeted molecular docking scores of selected LC–MS-annotated compounds against human acetylcholinesterase (PDB ID 7E3D).

Rank	Compound	Docking Score (kcal/mol)
1	6-hydroxy-2-(4-methoxyphenyl)-4H-chromen-4-one	−6.9
2	Campesterol	−6.8
2	Daidzein	−6.8
3	4-Hydroxy-3-methoxyphenylglycol	−5.2
3	Rosin	−5.0
4	1-Aminocyclopropane-1-carboxylic acid	−4.0

## Data Availability

The data that support the findings of this study are available from the corresponding author upon reasonable request.

## Supplementary Materials

The following supporting information can be downloaded at: https://www.mdpi.com/article/10.3390/molecules31203632/s1, Supplementary file: LCMS analysis of aqueous extract.
