# Supplementary material for: LC–MS-Based Metabolomic Profiling of Excoecaria agallocha Leaf Extract: Integrated Antioxidant, Anti-Inflammatory, Cholinesterase Inhibitory and Neuroprotective Evaluation with AChE-Targeted Molecular Docking
Source: Molecules. 2026 Oct 12;31(20):3632. doi: 10.3390/molecules31203632 (PMC13707391; doi:10.3390/molecules31203632)
Supplement: Supplementary file 1 [file molecules-31-03632-s001.zip › molecules-4586558-supplementary.pdf]

Supplementary file: LCMS analysis of aqueous extract

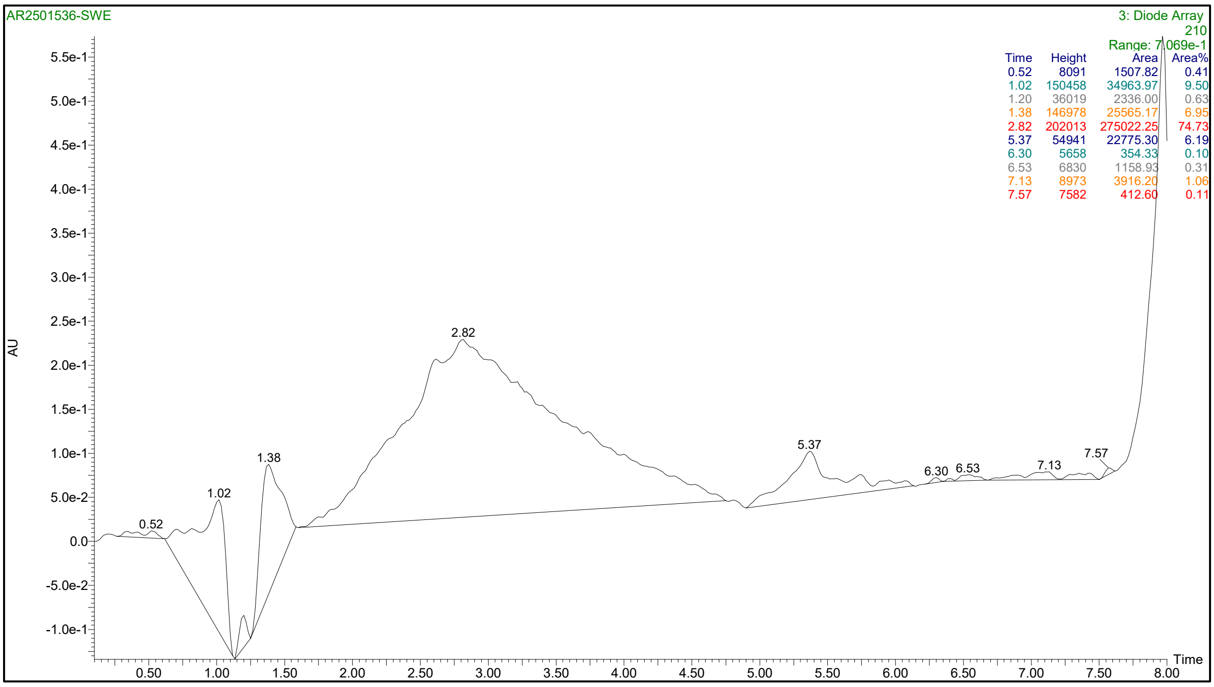

LC Chromatogram @210nm

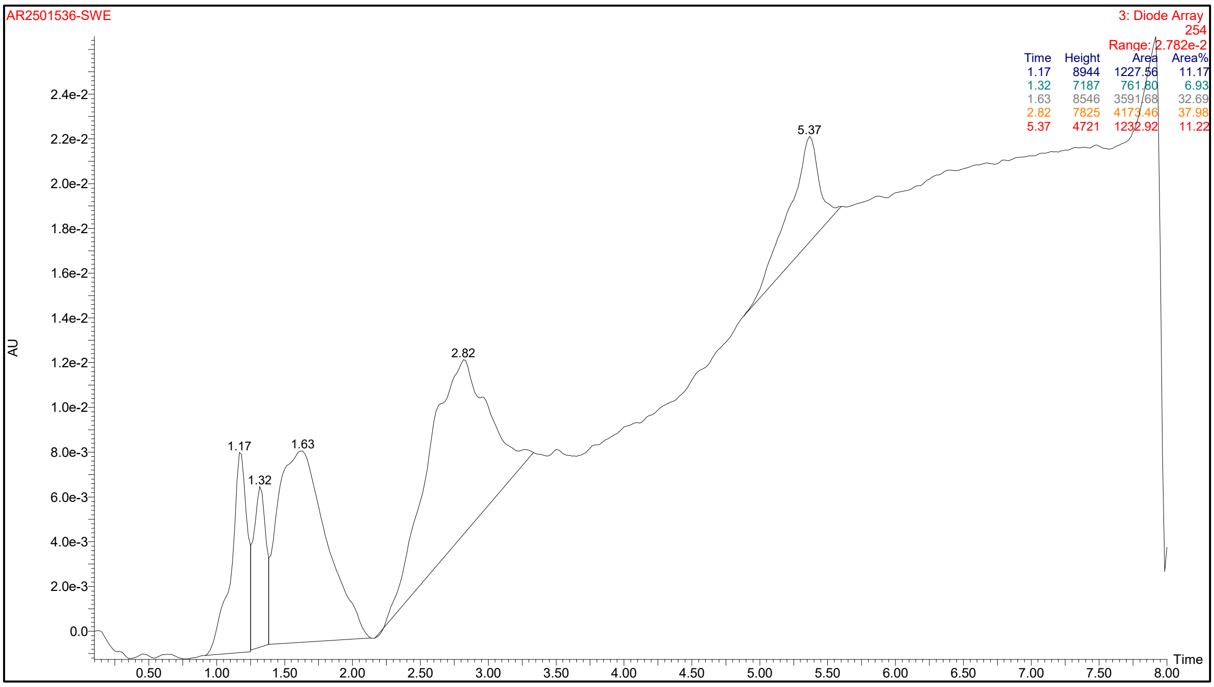

LC Chromatogram @254nm

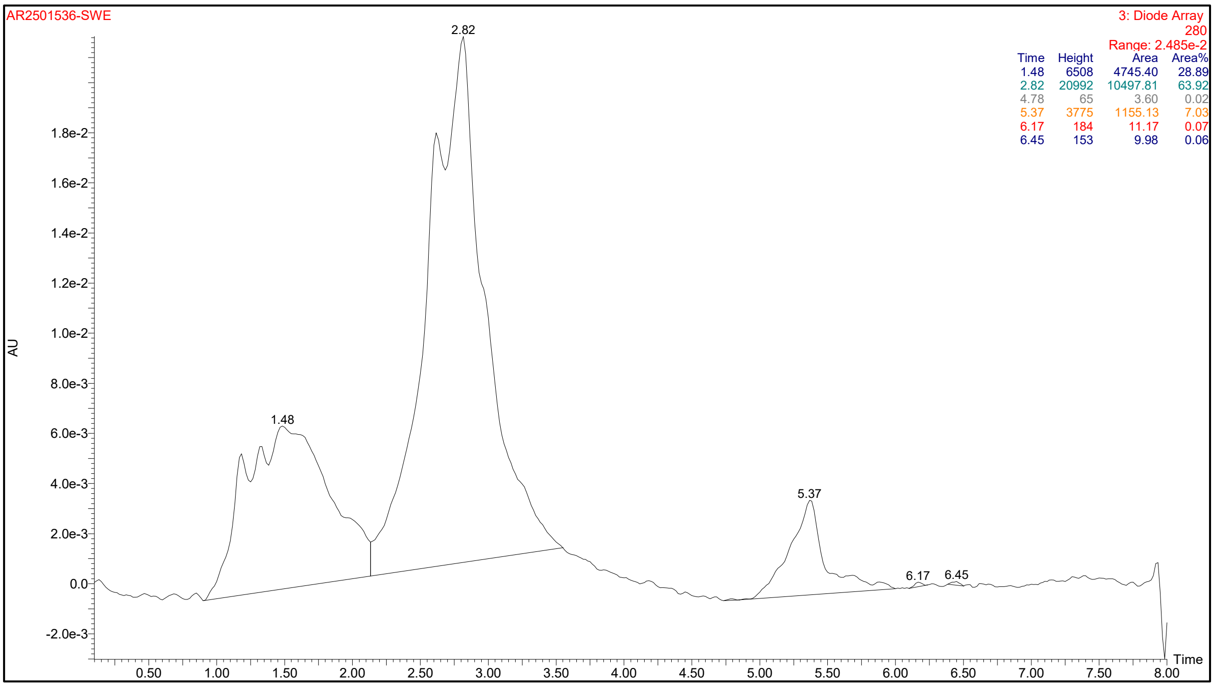

LC Chromatogram @280nm

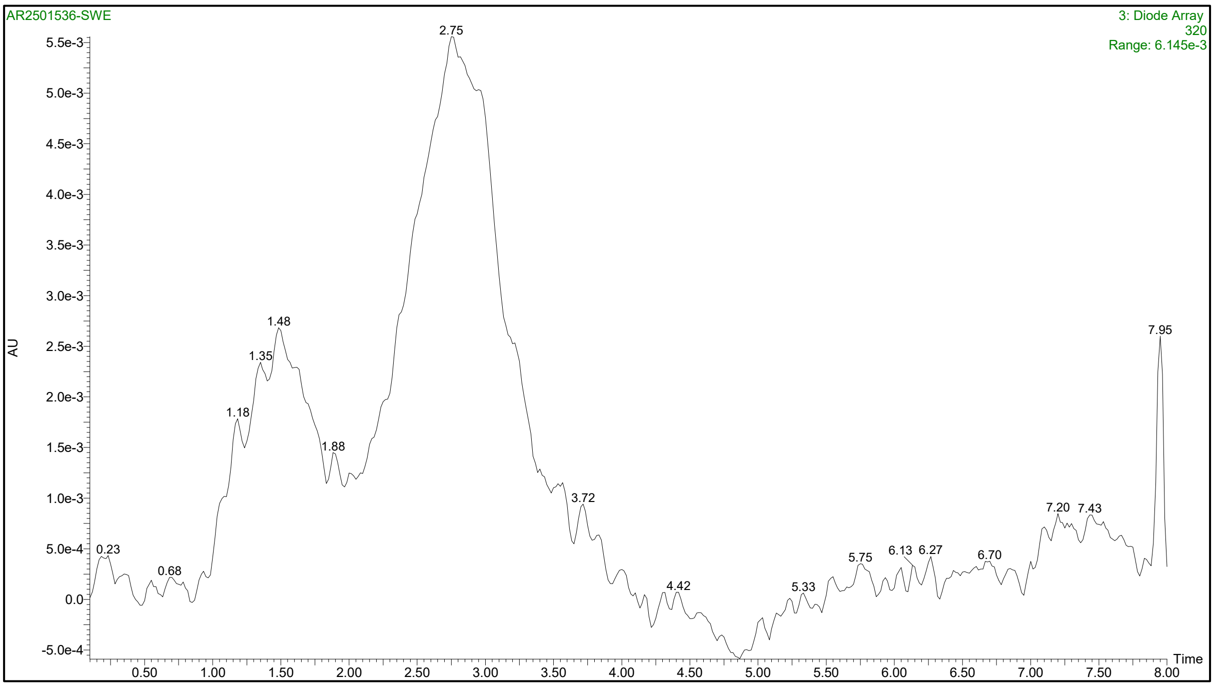

LC Chromatogram @320nm

LCMS Analysis:

Mobile Phase-A: 10mM Ammonium acetate in Water
Mobile Phase-B: Acetonitrile
Injection Volume: 10uL
Gradient Programme:

Time	Flow	%A	%B
Initial	1.00	90.0	10.0
3.00	1.00	2.0	98.0
6.00	1.00	2.0	98.0
6.50	1.00	90.0	10.0
8.00	1.00	90.0	10.0

LC Make: Waters, USA **Model:** 2695 Separations Module
MS Make: Waters, USA **Model:** Micromass ZQ
Column: Accucore C18, 50 x 4.6, 5µ Particle size from ThermoScientific.
Capillary Voltage: 2.8KV
Cone Volatage: 30V
Source Temp: 140°C
Desolvation Temp: 400°C
Desolvation Gas Flow: 600L/Hr
Processing Software: MassLynx V4.1

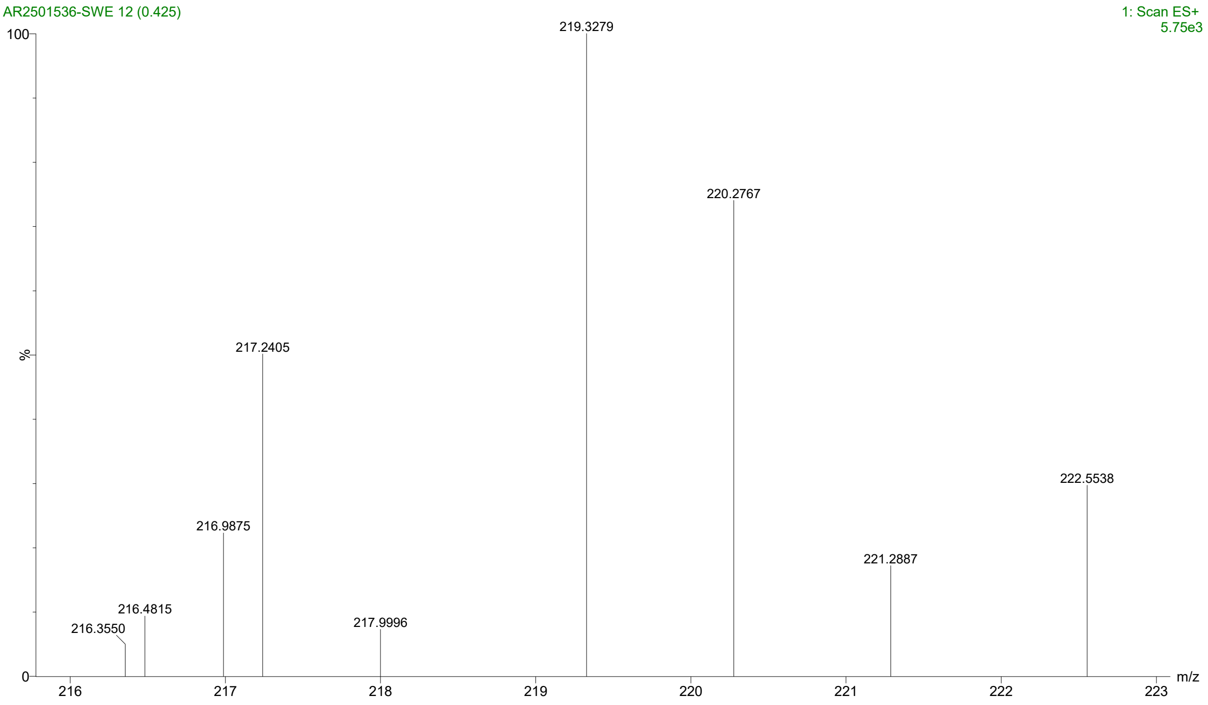

Mass Spectrum @RT 0.425

Name: 1-(2-(1H-indol-3-yl)ethyl)thiourea

Structure:

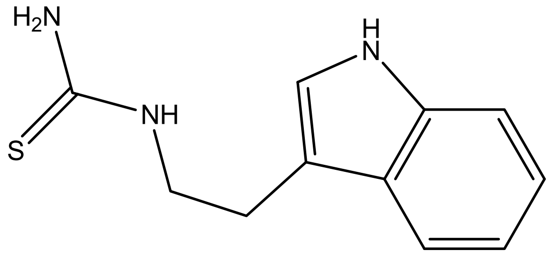

$C_{11}H_{13}N_3S$
Exact Mass: 219.083

Adduct: [M+H]+

Compound Class: 3-alkylindoles

InChIKey: HRBGONPRSGPFMT-UHFFFAOYSA-N

SMILES: S=C(N)NCCC1=CNC=2C=CC=CC21

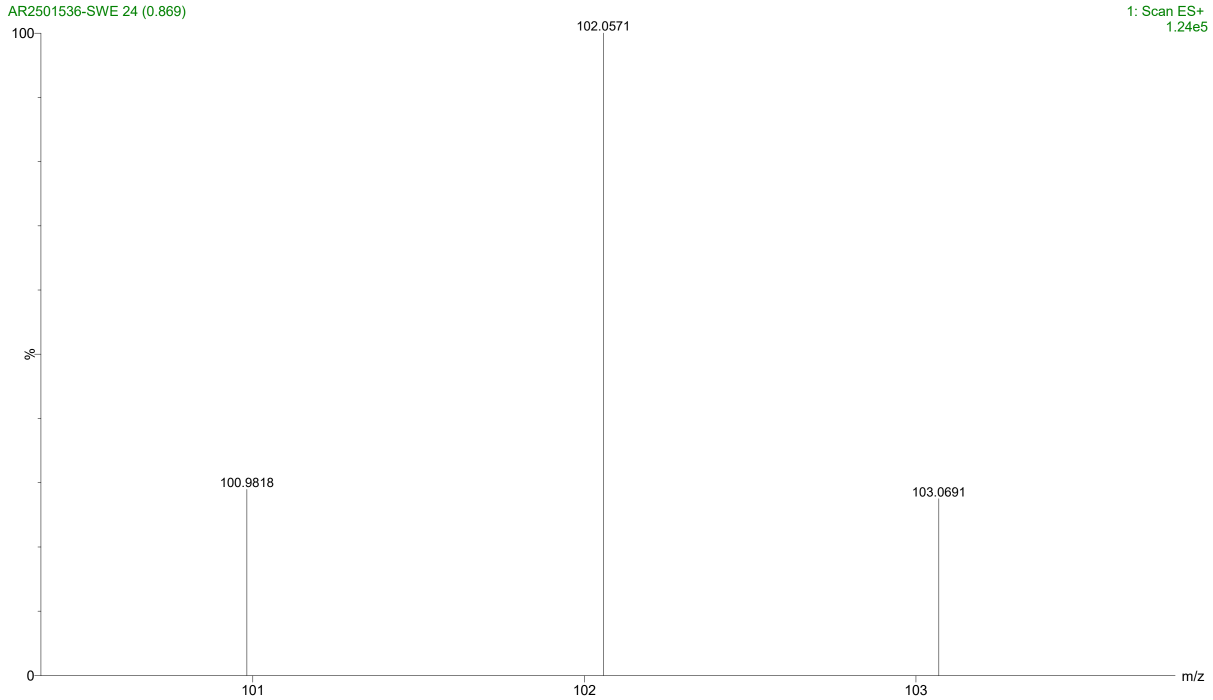

Mass Spectrum @RT 0.869

Name: 1-Aminocyclopropane-1-carboxylic acid
Structure:

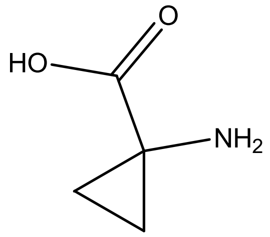

$C_4H_7NO_2$
Exact Mass: 101.0477

Adduct: [M+H]+
Compound Class: Alpha amino acids
InChIKey: PAJPWUMXBYXFCZ-UHFFFAOYSA-N
SMILES: OC(=O)C(N)(C1)C1

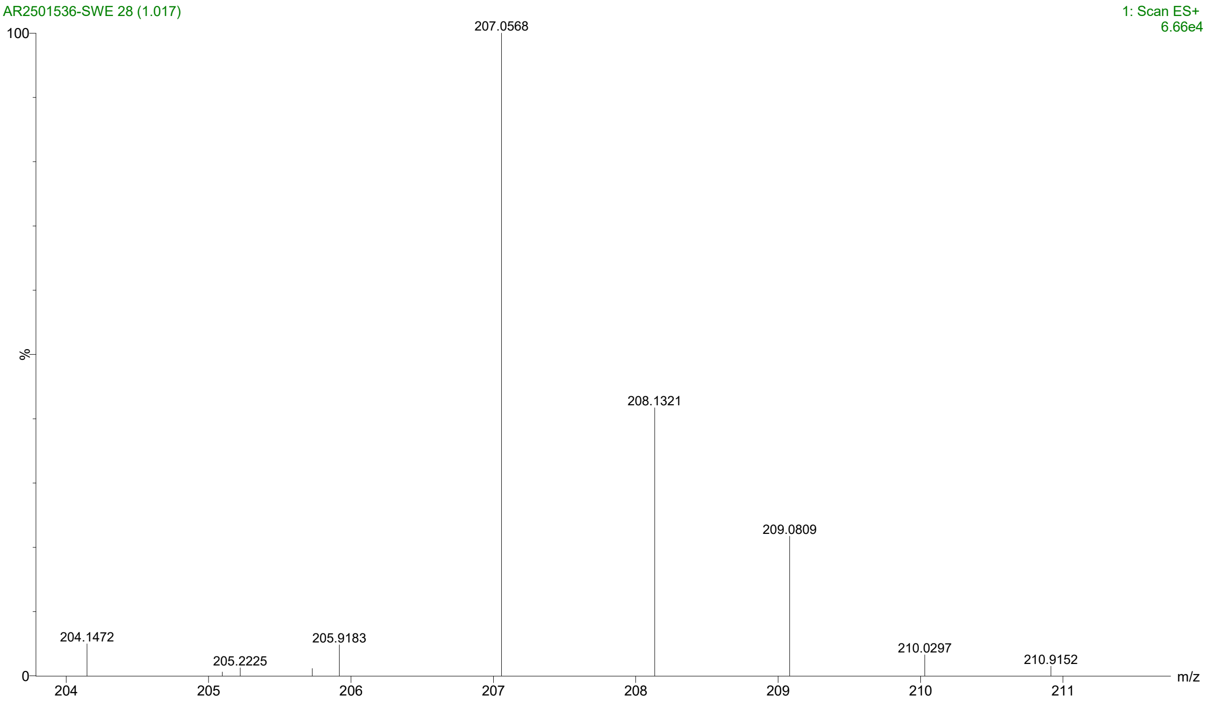

Mass Spectrum @RT 1.017

Name: 4-HYDROXY-3-METHOXYPHENYLGLYCOL

Structure:

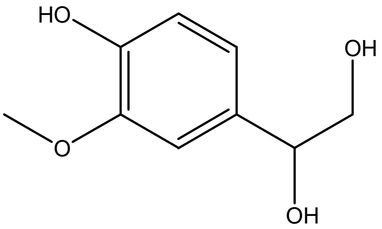

$C_9H_{12}O_4$
Exact Mass: 184.0736

Adduct: [M+Na]+

Compound Class: Methoxyphenols

InChIKey: FBWPWWWZWKPJFL-UHFFFAOYSA-N

SMILES: COC1=C(C=CC(=C1)C(CO)O)O

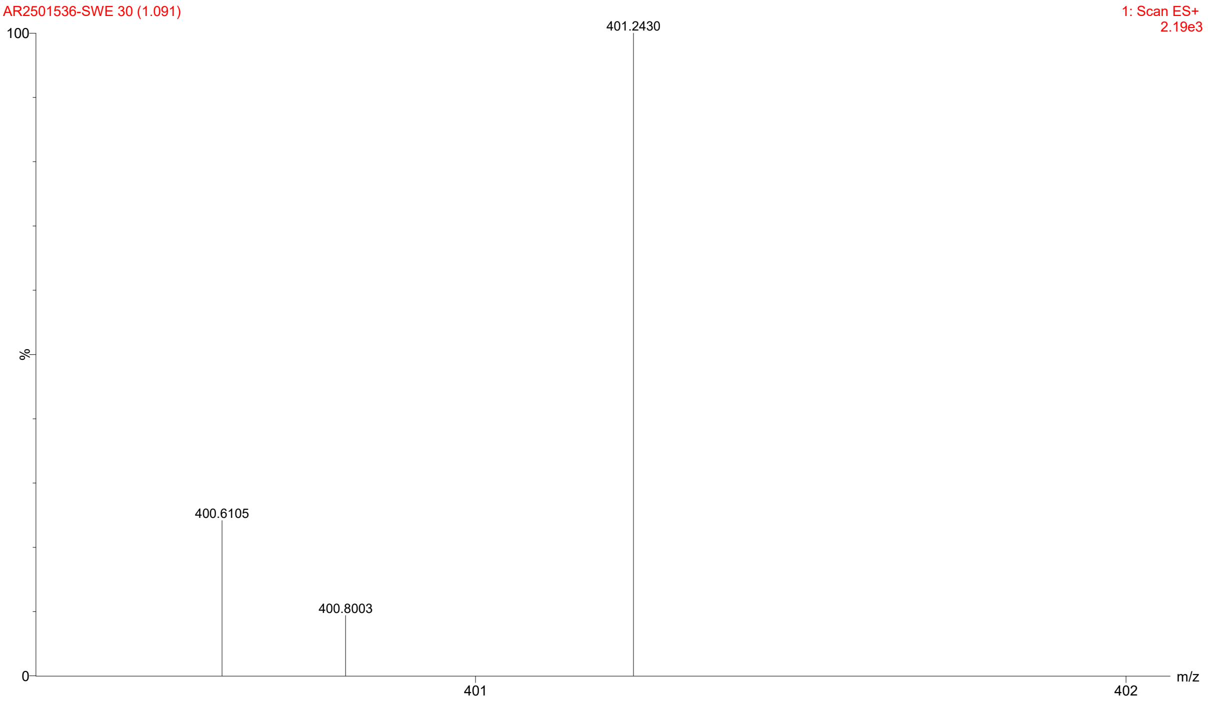

Mass Spectrum @RT 1.091

Name: Campesterol

Structure:

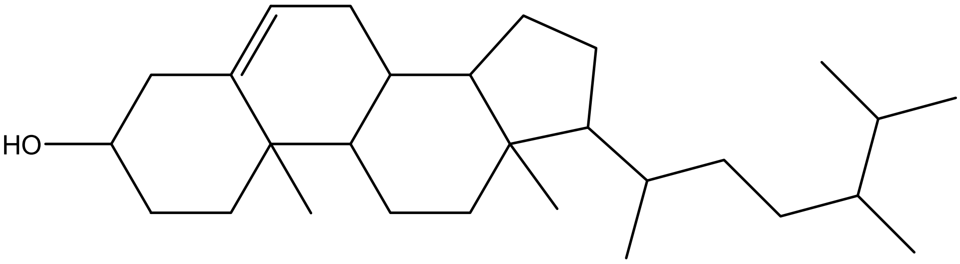

$C_{28}H_{48}O$
Exact Mass: 400.3705

Adduct: [M+H]+

Compound Class: Ergosterols and derivatives

InChIKey: SGNBVLSWZMBQTH-ZRUUVFCLSA-N

SMILES: OC1CC2=CCC3C(CCC4(C)C(CCC34)C(C)CCC(C)C(C)C)C2(C)CC1

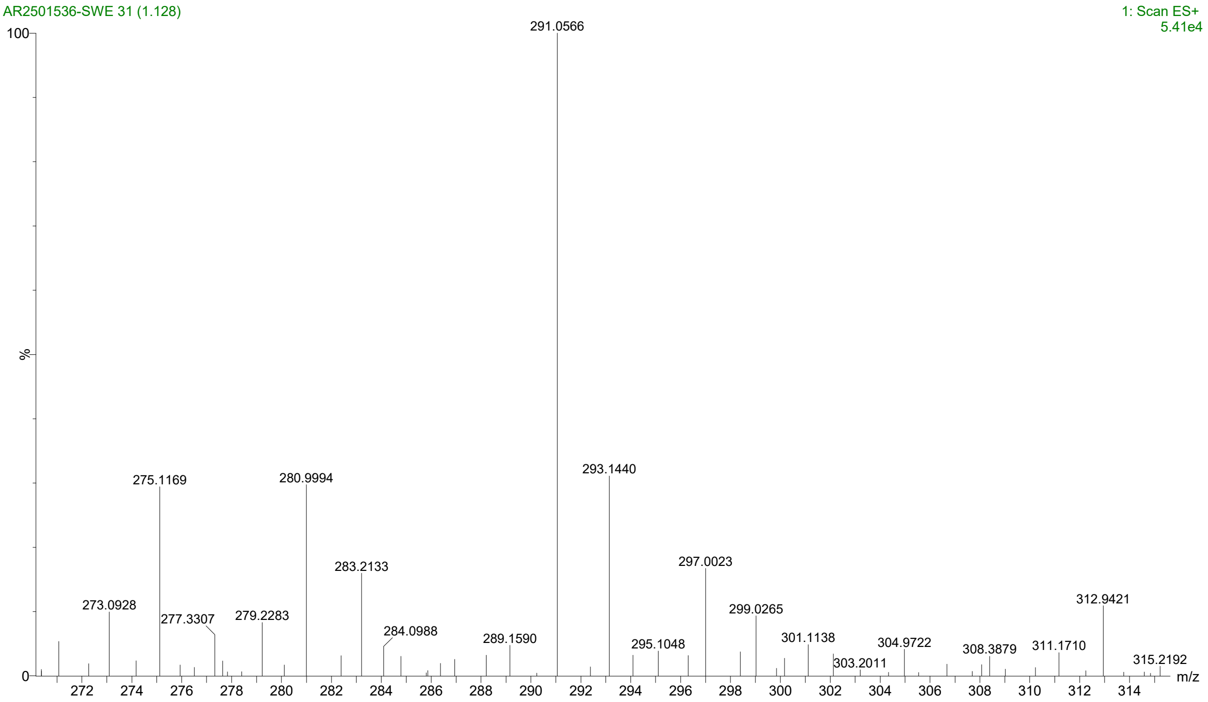

Mass Spectrum @RT 1.128

Name: 6-hydroxy-2-(4-methoxyphenyl)-4H-chromen-4-one

Structure:

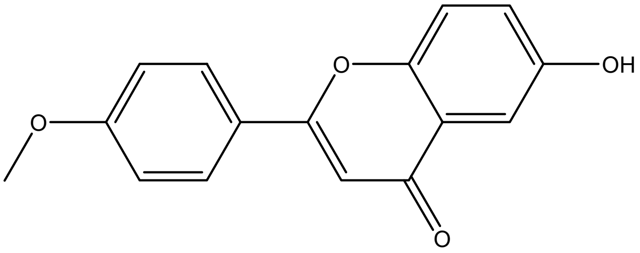

$C_{16}H_{12}O_4$
Exact Mass: 268.0736

Adduct: [M+Na]+

Compound Class: 4'-O-methylated flavonoids

InChIKey: RMPRESDCJMOAHW-UHFFFAOYSA-N

SMILES: O=C1C=C(OC2=CC=C(O)C=C12)C=3C=CC(OC)=CC3

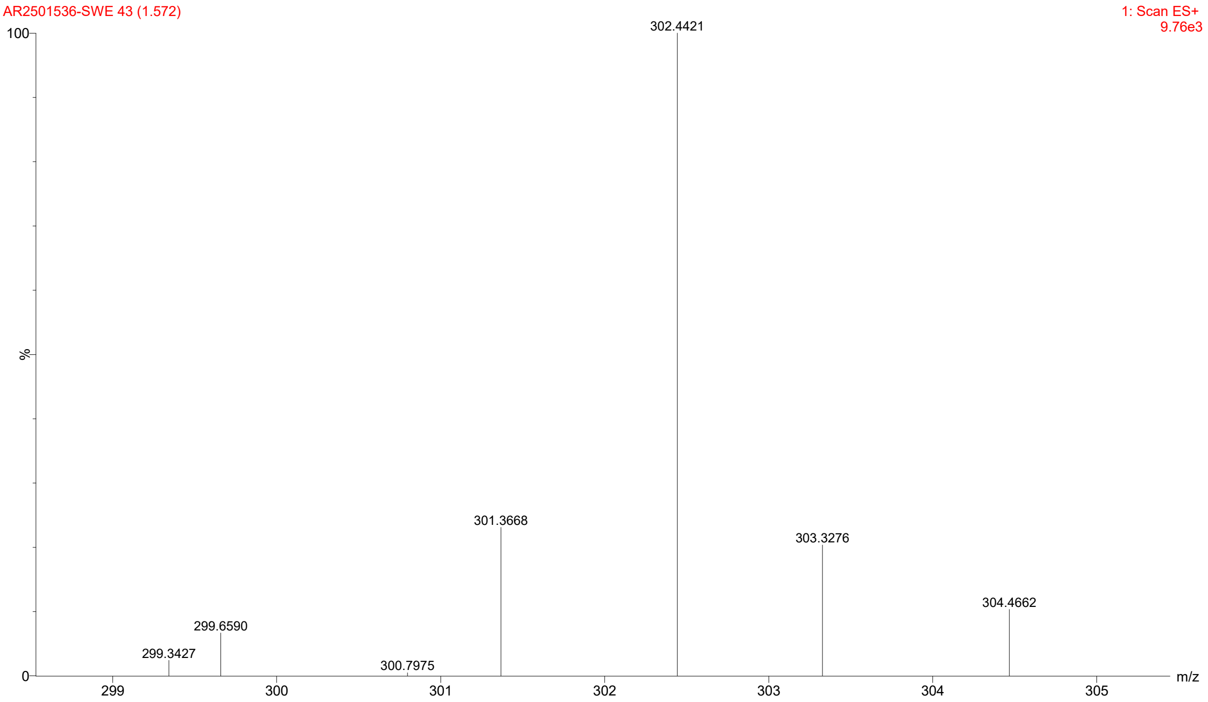

Mass Spectrum @RT 1.572

Name: D-erythro-Dihydrosphingosine

Structure:

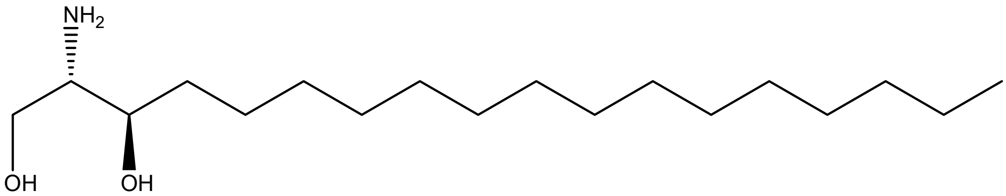

$C_{18}H_{39}NO_2$
Exact Mass: 301.2981

Adduct: [M+H]+

Compound Class: 1,2-aminoalcohols

InChIKey: OTKJDMGTUTTYMP-ZWKOTPCHSA-N

SMILES: CCCCCCCCCCCCCCC[C@@H](O)[C@@H](N)CO

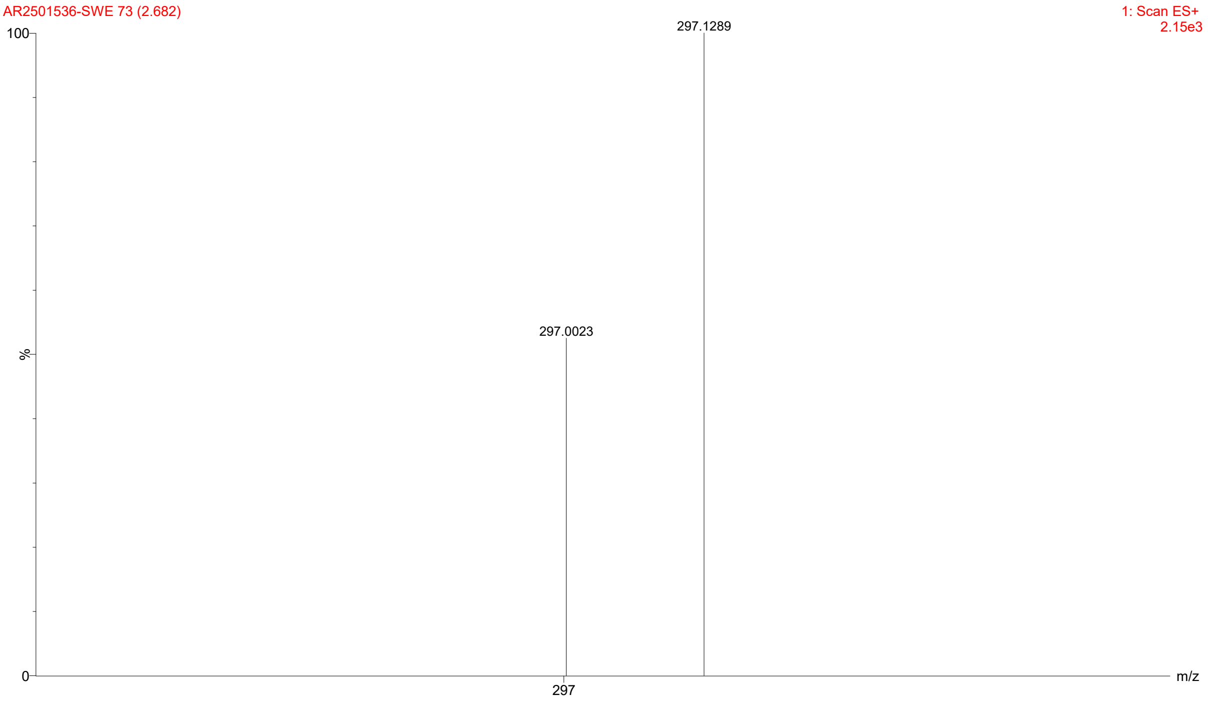

Mass Spectrum @RT 2.682

Name: Rosin

Structure:

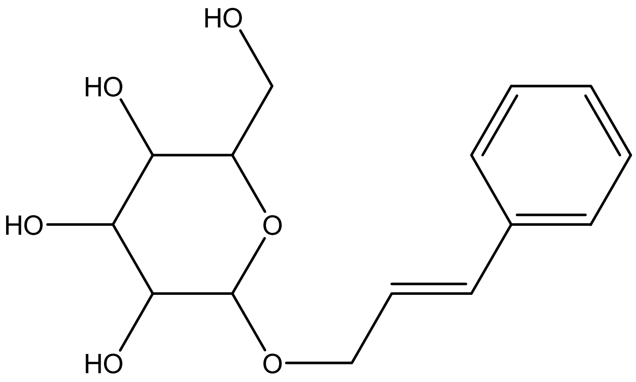

$C_{15}H_{20}O_6$
Exact Mass: 296.126

Adduct: [M+H]+

Compound Class: Fatty acyl glycosides of mono- and disaccharides

InChIKey: KHPCPRHQVVSZAH-GUNCLKARSA-N

SMILES: OCC1OC(OCC=CC=2C=CC=CC2)C(O)C(O)C1O

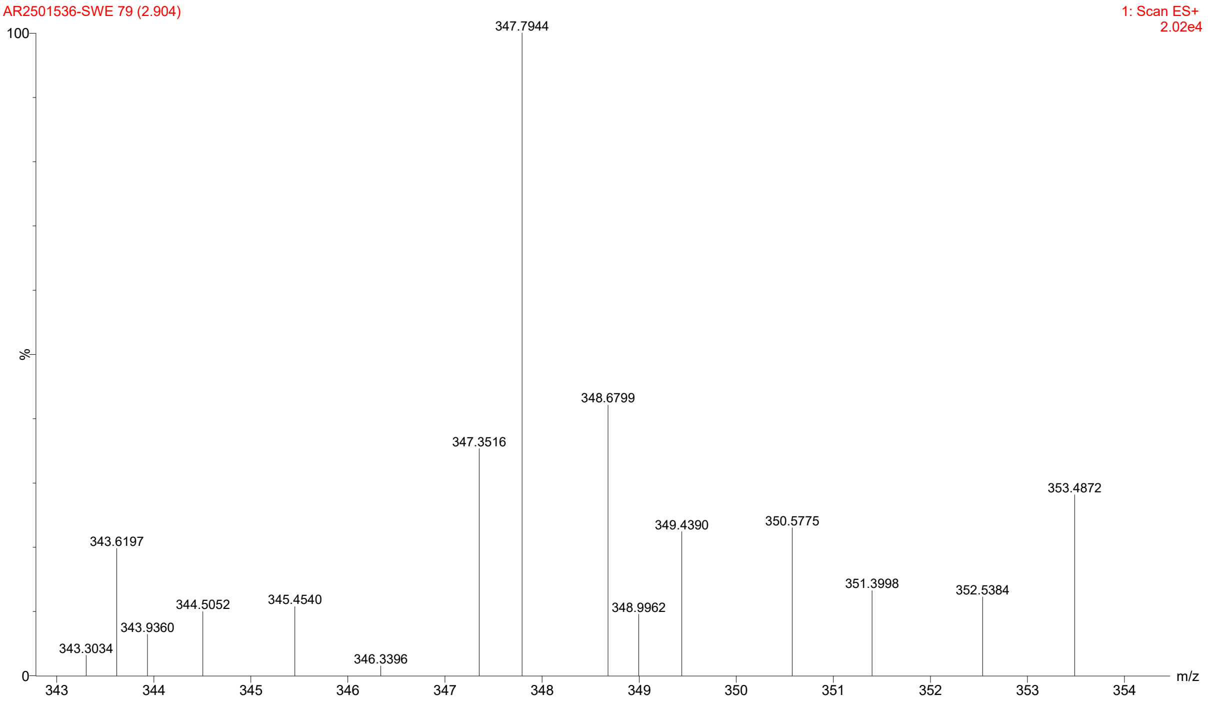

Mass Spectrum @RT 2.904

Name: 3-[(Z)-heptadec-10-enyl]benzene-1,2-diol

Structure:

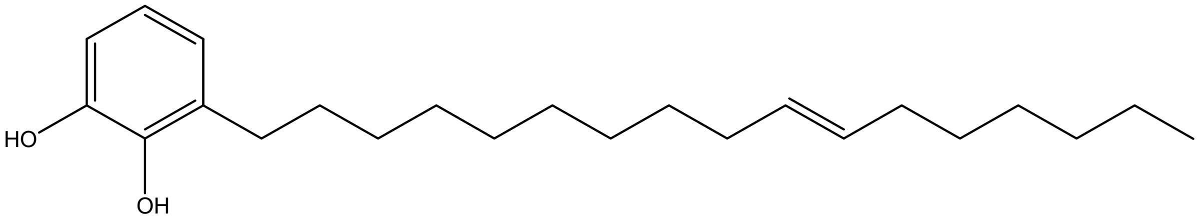

$C_{23}H_{38}O_2$
Exact Mass: 346.2872

Adduct: [M+H]+

Compound Class: Catechols

InChIKey: FNLMCNKPGGHKQL-FPLPWBNLSA-N

SMILES: OC1=CC=CC(=C1O)CCCCCCCCCC=CCCCCCC

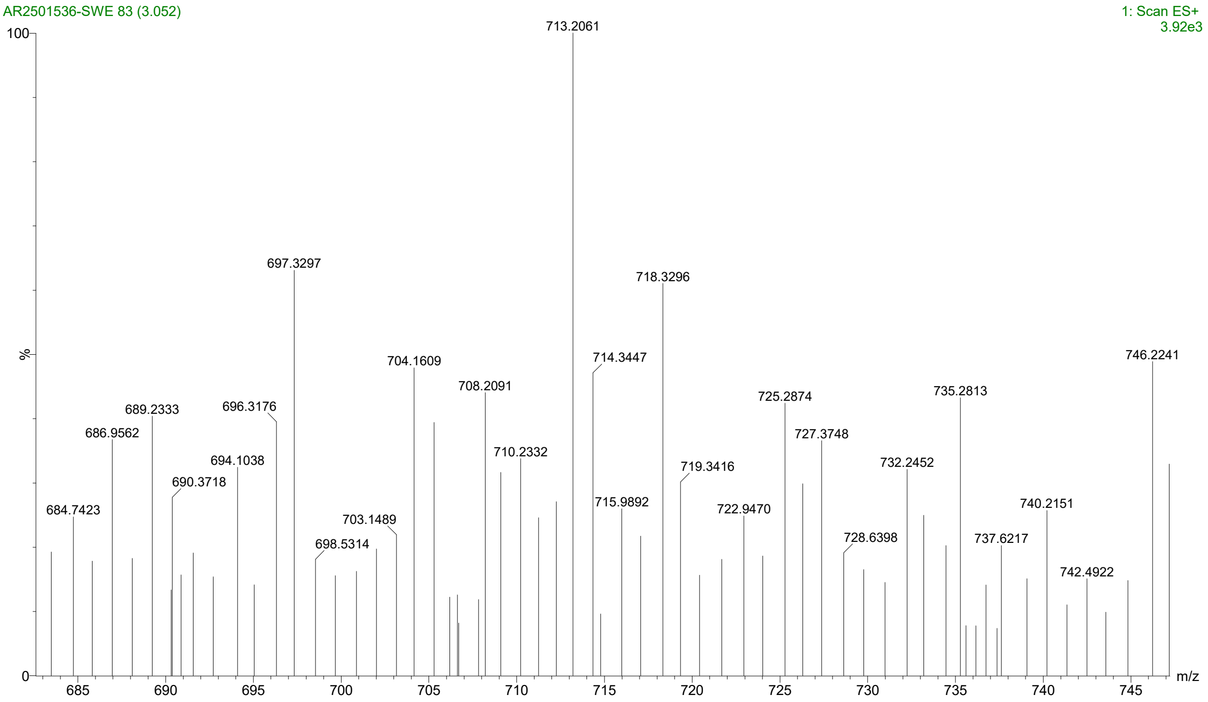

Mass Spectrum @RT 3.052

Name: Syringaresnol-4-O-beta-D-apiofuranosy

Structure:

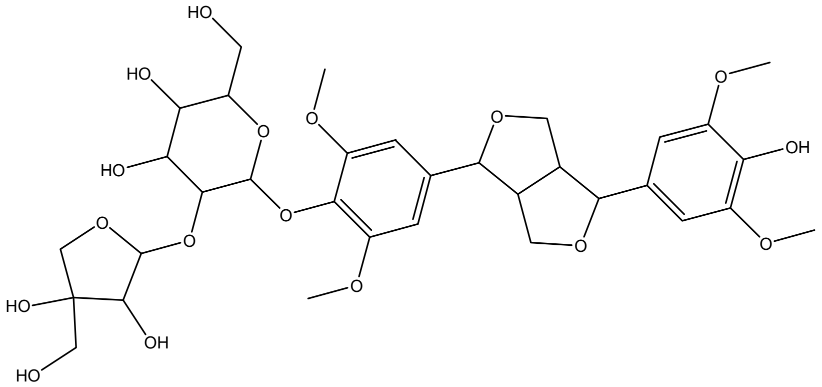

$C_{33}H_{44}O_{17}$
Exact Mass: 712.2578

Adduct: [M+H]+

Compound Class: Lignan glycosides

InChIKey: BUBVKRMIMSPLND-XSTKEGOHSA-N

SMILES:

OC1=C(OC)C=C(C=C1OC)C2OCC3C(OCC23)C4=CC(OC)=C(OC5OC(CO)C(O)C(O)C5OC6OCC(O)(CO)C6O)C(OC)=C4

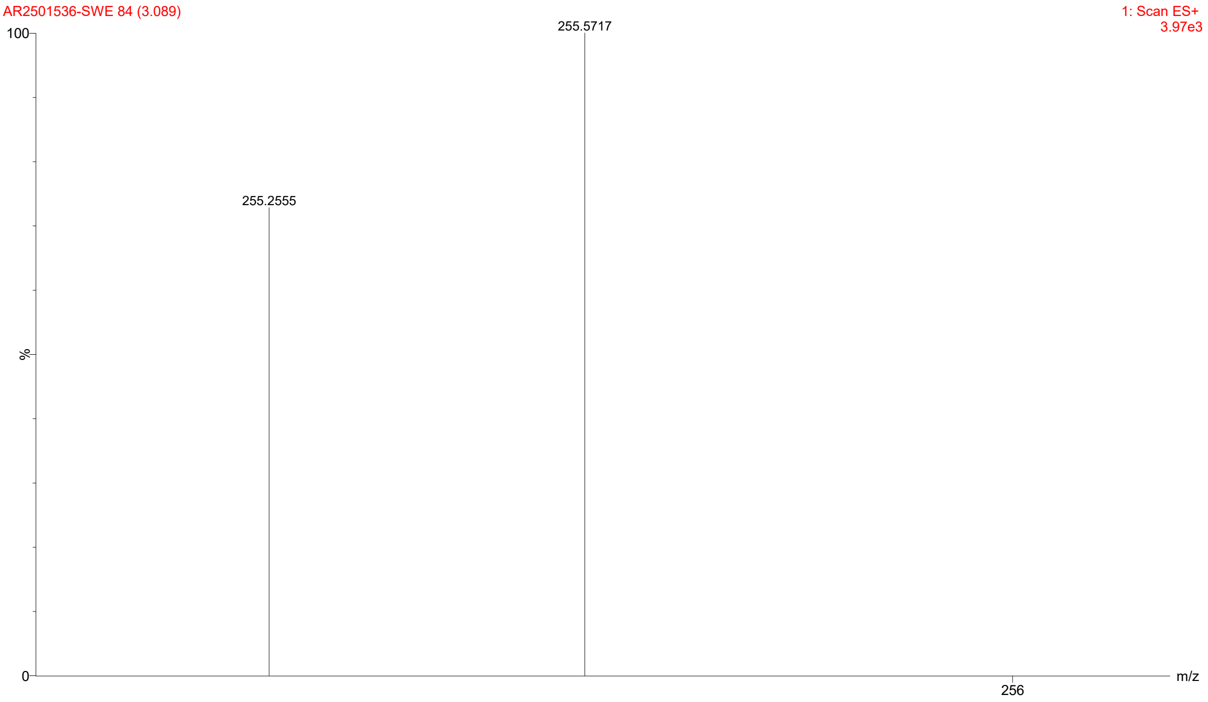

Mass Spectrum @RT 3.089

Name: Daidzein

Structure:

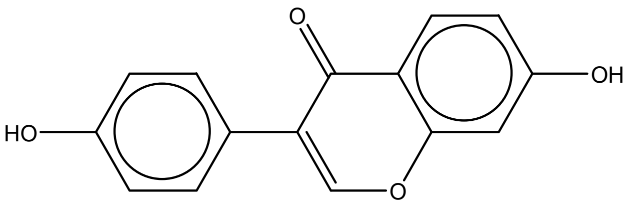

$C_{15}H_{10}O_4$
Exact Mass: 254.0579

Adduct: [M+H]+

Compound Class: Isoflavones

InChIKey: ZQSIJRDFPHDXIC-UHFFFAOYSA-N

SMILES: Oc(c3)ccc(c3)C(=C1)C(=O)c(c2)c(cc(O)c2)O1

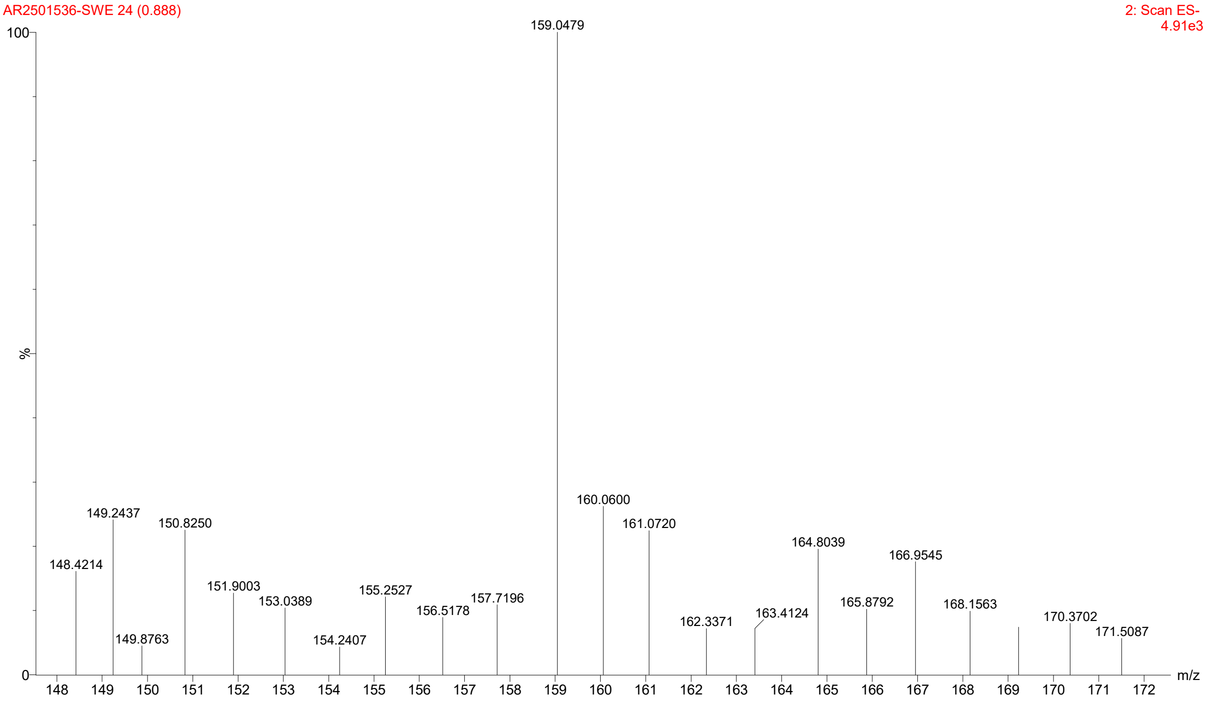

Mass Spectrum @RT 0.888

Name: 1-Aminocyclopropane-1-carboxylic acid

Structure:

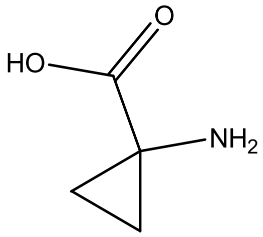

$C_4H_7NO_2$

Exact Mass: 101.0477

Adduct: [M+H]+

Compound Class: Alpha amino acids

InChIKey: PAJPWUMXBYXFCZ-UHFFFAOYSA-N

SMILES: OC(=O)C(N)(C1)C1

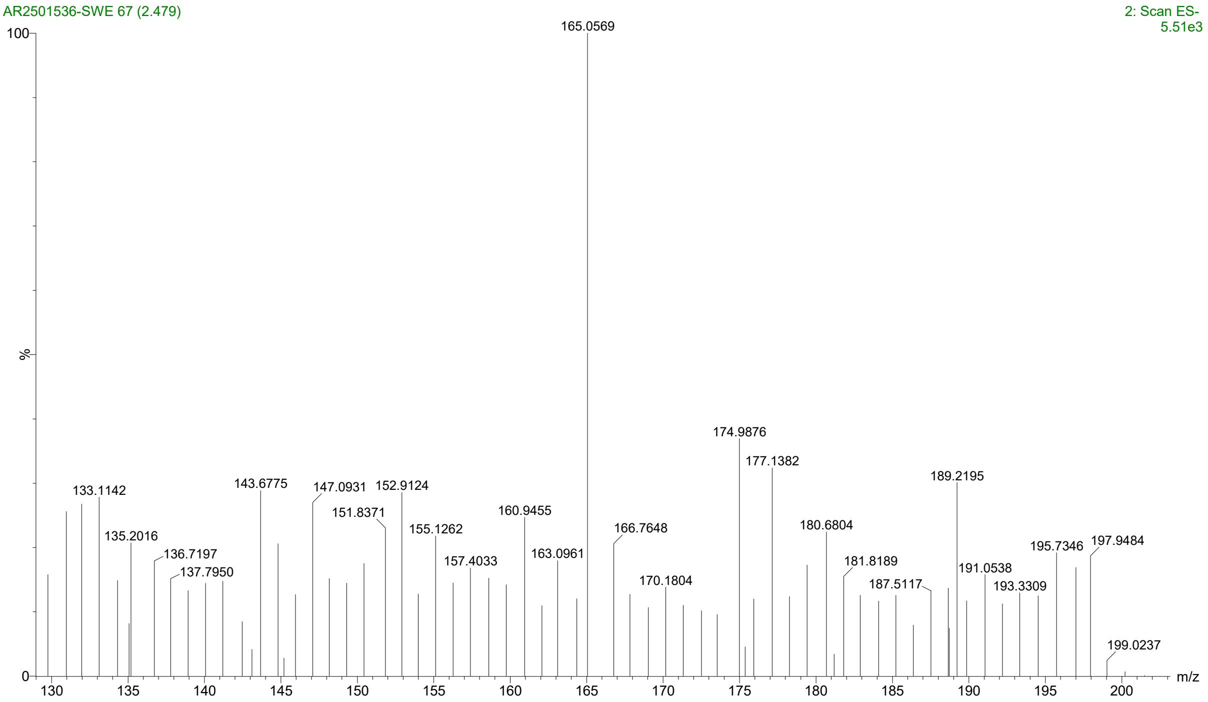

Mass Spectrum @RT 2.479

Name: 3-(2-HYDROXYPHENYL) PROPANOATE

Structure:

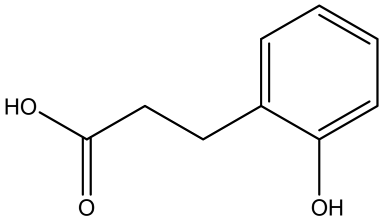

$C_9H_{10}O_3$
Exact Mass: 166.063

Adduct: [M+H]+

Compound Class: Phenylpropanoic acids

InChIKey: CJBDUOMQLFKVQC-UHFFFAOYSA-N

SMILES: OC(=O)CCC1=CC=CC=C1O
